# Rescue in vitro maturation using ovarian support cells of human oocytes from conventional stimulation cycles yields oocytes with improved nuclear maturation and transcriptomic resemblance to in vivo matured oocytes

**DOI:** 10.1007/s10815-024-03143-4

**Published:** 2024-05-30

**Authors:** Bruna Paulsen, Sabrina Piechota, Ferran Barrachina, Alexa Giovannini, Simone Kats, Kathryn S. Potts, Graham Rockwell, Maria Marchante, Samantha L. Estevez, Alexander D. Noblett, Alexandra B. Figueroa, Caroline Aschenberger, Dawn A. Kelk, Marcy Forti, Shelby Marcinyshyn, Klaus Wiemer, Marta Sanchez, Pedro Belchin, Joseph A. Lee, Erkan Buyuk, Rick E. Slifkin, Merrick Pierson Smela, Patrick R. J. Fortuna, Pranam Chatterjee, David H. McCulloh, Alan B. Copperman, Daniel Ordonez-Perez, Joshua U. Klein, Christian C. Kramme

**Affiliations:** 1 Gameto Inc., 430 E. 29th St Fl 14, New York, NY 10016 USA; 2 Obstetrics, Gynecology, and Reproductive Science, Icahn School of Medicine at Mount Sinai, New York, NY USA; 3 Extend Fertility, New York, NY USA; 4 KEW Technology, Seattle, WA USA; 5 Ruber Juan Bravo University Hospital, Eugin Group, Madrid, Spain; 6 Reproductive Medicine Associates of New York, New York, NY USA; 7 Wyss Institute, Harvard Medical School, Boston, MA USA; 8 Department of Genetics, Harvard Medical School, Boston, MA USA; 9 Department of Biomedical Engineering, Duke University, Durham, NC USA; 10 Department of Computer Science, Duke University, Durham, NC USA

**Keywords:** Ovarian support cells, In vitro maturation, Stem cells, Oocyte transcriptomics, Granulosa cells

## Abstract

**Purpose:**

Determine if the gene expression profiles of ovarian support cells (OSCs) and cumulus-free oocytes are bidirectionally influenced by co-culture during in vitro maturation (IVM).

**Methods:**

Fertility patients aged 25 to 45 years old undergoing conventional ovarian stimulation donated denuded immature oocytes for research. Oocytes were randomly allocated to either OSC-IVM culture (intervention) or Media-IVM culture (control) for 24–28 h. The OSC-IVM culture condition was composed of 100,000 OSCs in suspension culture with human chorionic gonadotropin (hCG), recombinant follicle stimulating hormone (rFSH), androstenedione, and doxycycline supplementation. The Media-IVM control lacked OSCs and contained the same supplementation. A limited set of in vivo matured MII oocytes were donated for comparative evaluation. Endpoints consisted of MII formation rate, morphological and spindle quality assessment, and gene expression analysis compared to in vitro and in vivo controls.

**Results:**

OSC-IVM resulted in a statistically significant improvement in MII formation rate compared to the Media-IVM control, with no apparent effect on morphology or spindle assembly. OSC-IVM MII oocytes displayed a closer transcriptomic maturity signature to IVF-MII controls than Media-IVM control MII oocytes. The gene expression profile of OSCs was modulated in the presence of oocytes, displaying culture- and time-dependent differential gene expression during IVM.

**Conclusion:**

The OSC-IVM platform is a novel tool for rescue maturation of human oocytes, yielding oocytes with improved nuclear maturation and a closer transcriptomic resemblance to in vivo matured oocytes, indicating a potential enhancement in oocyte cytoplasmic maturation. These improvements on oocyte quality after OSC-IVM are possibly occurring through bidirectional crosstalk of cumulus-free oocytes and ovarian support cells.

**Supplementary Information:**

The online version contains supplementary material available at 10.1007/s10815-024-03143-4.

## Introduction

Oocyte maturation is a synchronized nuclear and cytoplasmic developmental process that results in the extrusion of the first polar body (PB1) and deposition of proteins, organelles, and transcripts needed for fertilization competence and embryogenesis [1, 2]. Much of this developmental process is coordinated by several lineages of ovarian somatic cell types including stroma, theca, and granulosa cells, which send critical developmental signals to oocytes and supporting cells through paracrine and autocrine mechanisms [3, 4]. It is well known that during maturation, oocytes are largely transcriptionally silent, relying on post-transcriptional modification, transcript degradation, and selective translation to control cell cycle and development until zygotic genome activation [5–7]. Few studies have evaluated transcriptome-wide changes in human oocyte gene expression following in vitro maturation (IVM), particularly in response to different methods of IVM, which may differ in their mechanism of action [8–10]. Additionally, there is limited understanding of how somatic support cells, besides cumulus cells, can influence the gene expression and development of human oocytes matured in vitro.

Although immature oocytes removed from the follicular environment can undergo meiosis, their rate of maturation and subsequent developmental competence has not been sufficiently reliable for widespread clinical use [1, 11]. Decades of research into how oocytes develop have led to the creation of IVM-stimulating cell culture media, which are commercially available for human oocyte IVM. Such IVM media products are usually designed for oocytes enclosed in cumulus cells, limiting their utility for denuded immature oocyte rescue applications [12–23]. While the primary clinical benefit of IVM is to reduce gonadotropin usage in stimulation and provide safe treatment options for women contraindicated to conventional controlled ovarian stimulation (COS), for women whose oocytes are all or mostly immature at retrieval after traditional COS, solutions for effective rescue IVM may allow for successful treatment [24, 25]. Limited studies have shown that denuded oocyte maturation can be coordinated and improved by co-cultured primary granulosa cells and other follicle-mimicking interventions, indicating these oocytes may be directly responsive to extrinsic paracrine signaling despite their lack of cumulus cells and direct cell junctions [26, 27]. However, few studies have investigated how aspects of cytoplasmic maturation such as oocyte transcript abundance change in response to in vitro signaling modalities, and few rescue IVM solutions show clinical promise for their ability to yield high-quality mature oocytes [20, 28, 29]. Therefore, investigation is needed into understanding how the oocyte transcriptome is influenced by the IVM environment, and innovative solutions are needed to develop efficacious rescue IVM technologies.

Our group has recently demonstrated a novel technology to generate ovarian support cells (OSCs) from human-induced pluripotent stem cells (hiPSCs) in a rapid, efficient, and reproducible manner through transcription factor (TF)-directed differentiation [30]. The OSCs are composed of FOXL2 + AMHR2 + NR2F2 + granulosa-like cells. These cells are growth factor producing and in the presence of follicle stimulating hormone (FSH) are steroidogenic [30]. We have recently demonstrated the potential of hiPSC-derived OSCs to increase in vitro oocyte maturation and euploid embryo formation rates from abbreviated gonadotropin cycles [31]. In this study, we further explored the potential of hiPSC-derived OSCs to rescue immature denuded human oocytes retrieved in conventional COS cycles, and we determined how gene expression is modulated in oocytes and OSCs by OSC-IVM co-culture.

## Materials and methods

### Collection of immature oocytes

#### Subject ages, IRB, and informed consent

Forty-seven fertility patient subjects were enrolled in the study for donating oocytes for research purposes using informed consent (IRB# 1345762, Western IRB and CNRHA 47/428973.9/22). Subject ages were between 25 and 45 years of age, with an average age of 35. Exclusion criteria included donors with a history of diabetes, thyroid disease, endometriosis, history of recurrent implantation failure, and known history of oocyte maturation defect or chromosomal abnormalities. A limited cohort of pre-vitrified immature oocytes and in vivo matured oocytes which were banked for research purposes under informed consent were donated for spindle and transcriptomic analysis (IRB# 1332581, Western IRB).

#### Oocyte retrieval and donation

Fertility patients providing discarded immature oocytes had signed informed consents, provided by the clinic, permitting their use for research purposes. Patients underwent typical age-appropriate controlled ovarian stimulation using gonadotropin-releasing hormone (GnRH) analogs (agonist or antagonist) and injections with recombinant or highly purified urinary gonadotropins (recombinant FSH, human menopausal gonadotropins) followed by an ovulatory trigger (human chorionic gonadotropin (hCG) or GnRH agonist). Thirty-four to thirty-six hours following the trigger injection(s), oocytes were retrieved from the patient under conscious sedation using standard clinical procedures.

**Fig. 1:**
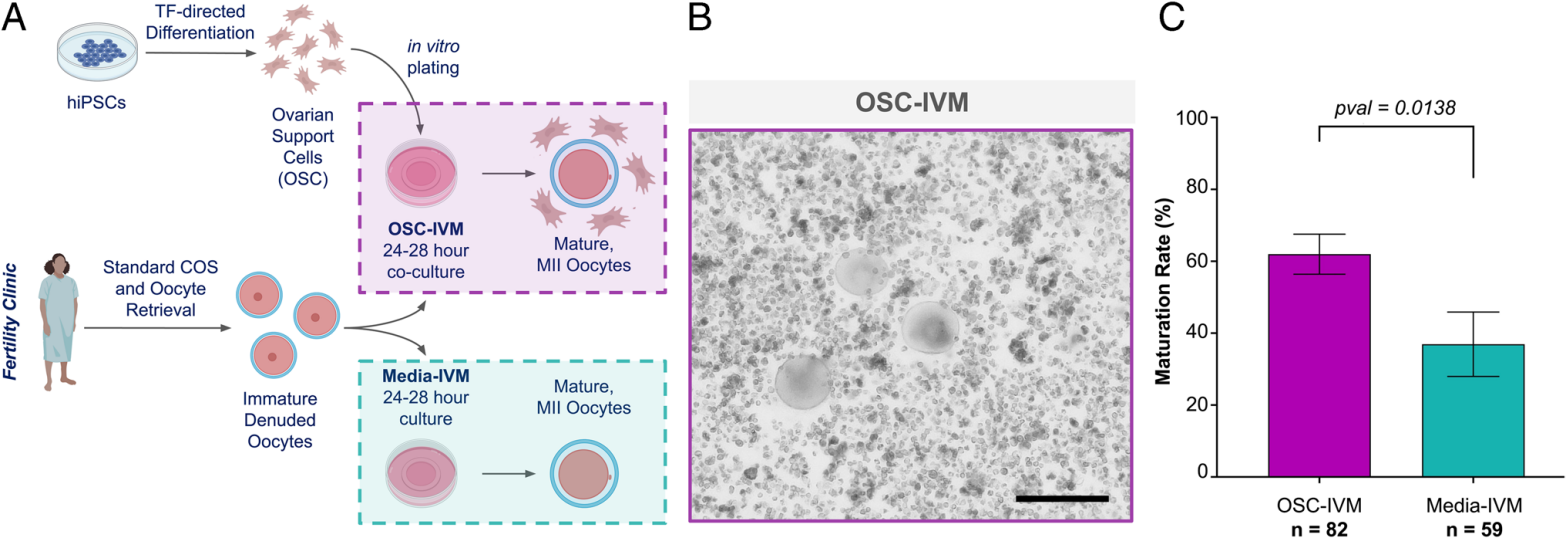
Treatment with OSC-IVM improves maturation rate of human denuded oocytes compared to an IVM media-matched control. **A** Schematic of the experimental co-culture IVM approach. hiPSCs were differentiated using inducible transcription factor overexpression to form ovarian support cells (OSCs). Human oocytes were obtained from patients in the clinic after standard gonadotropin stimulation, and immature oocytes (GV and MI) identified after denuding were allocated between the experimental OSC-IVM condition (OSC-IVM) or the control IVM media condition (Media-IVM) for IVM co-culture. Oocyte maturation and health were assessed after 24–28 h IVM co-culture, and oocytes were frozen for further analyses. The figure was created with BioRender.com. **B** Representative image of co-culture containing immature human oocytes (*n* = 3) and OSCs. Scale bar: 200 µm. Denuded GV oocytes are seen with surrounding OSCs in suspension culture. **C** Maturation rate of oocytes after 24–28 h IVM experiments, including oocyte co-culture with OSCs (OSC-IVM), or in media control (Media-IVM). *n* indicates the number of individual oocytes in each culture condition. Error bars indicate mean ± SEM. The *p*-value is derived from unpaired *t*-test comparing experimental OSC-IVM to control Media-IVM. Due to low numbers of retrieved oocytes per donor, each group contains oocytes from predominantly non-overlapping donor groups, and pairwise comparisons are not utilized

Retrieved oocytes were exposed to hyaluronidase briefly then adherent cumulus cells were mechanically removed by repeatedly drawing up and expelling each cumulus-oocyte complex with a small-bore pipette. Denuded oocytes were assessed for maturation by observation of a polar body or a germinal vesicle (GV). Immature oocytes, GV or Metaphase I (MI), which would usually be discarded, were instead allocated to the research study (Supplementary Fig. 1). All immature oocytes retrieved from the clinic each day were pooled and were placed into a pre-maturation LAG Medium (Medicult, Cooper Surgical) in a 5 ml round-bottom tube that was transferred from the clinic to the research laboratory in a 37 °C transport incubator. Upon arrival at the research facility, oocytes were placed to the corresponding in vitro maturation (IVM) condition following randomized sibling oocyte allocation. After IVM, oocyte maturation and total oocyte score (TOS) were assessed, and oocytes were snap-frozen for subsequent RNA sequencing analysis. All oocytes used for evaluation of nuclear maturation rate (Fig. 1) were fresh and subjected to IVM the day of oocyte retrieval.

For spindle assembly assessment experiments, retrieved immature (GV and MI) oocytes were vitrified or slow-frozen and stored at the clinic. Cryopreserved oocytes were transported from the clinic to the laboratory in liquid nitrogen and stored until use. Oocytes were then thawed using the standard Kitazato protocol for vitrified or slow-frozen oocytes (Kitazato, USA and Vitrolife, USA), and immature oocytes (GV or MI) were then allocated to the corresponding IVM condition following randomized sibling oocyte allocation and utilized for spindle position evaluation (Supplementary Fig. 1). All immature oocytes were placed into the IVM conditions in an identical manner, independent of the clinical donation source.

A limited number of metaphase II (MII) oocytes obtained from conventional controlled ovarian stimulation, which were previously banked for research purposes, were provided as controls for this study (IVF-MII). These oocytes were transferred to our laboratory and thawed using either the standard Kitazato protocol for vitrified oocytes (Kitazato, USA) or slow freeze–thaw protocol for previously slow frozen oocytes (Vitrolife, USA), and utilized for spindle position evaluation and TOS (Supplementary Fig. 1). Oocytes used for transcriptomic analysis were snap-frozen (Supplementary Fig. 1).

### Preparation of ovarian support cells (OSCs)

Human-induced pluripotent stem cell (hiPSC)-derived OSCs were created according to the transcription factor (TF)-directed protocol previously described [30]. In short, modified hiPSCs harboring specific TF expression vectors were induced for 5 days with doxycycline to trigger overexpression of TFs (*NR5A1, RUNX2, GATA4*) and drive differentiation into OSCs. For the initial 2 days of the differentiation protocol, hiPSCs were treated with CHIR99021 and Y-27632 to support mesodermal induction. OSCs generated for this study were derived from F66 parental hiPSC line, and therefore, the treatment was allogeneic to the oocyte donor samples. OSCs were produced in multiple batches and cryopreserved in vials of 120,000 to 150,000 cells each and stored in the vapor phase of liquid nitrogen in CryoStor CS10 Cell Freezing Medium (StemCell Technologies). A post-thaw flow cytometry assessment using the CD82 granulosa cell marker was conducted to validate OSC identity, with a criterion set at > 80% of CD82 + live cells.

Culture dishes (4 + 8 Well Dishes, BIRR) for oocyte maturation experiments were prepared with culture media and additional constituents in 100 µl droplets under mineral oil (LifeGuard, LifeGlobal Group) the day before oocyte collection and equilibrated in the incubator overnight. The morning of oocyte collection, cryopreserved OSCs were thawed for 2–3 min at 37 °C (in a heated bead or water bath), resuspended in OSC-IVM medium (Table 1), and washed twice using centrifugation and pelleting to remove residual cryoprotectant. Equilibrated OSC-IVM medium was used for final cell resuspension. OSCs were then plated at a concentration of 100,000 OSCs per 100 µl droplet by replacing 50 µl of the droplet with 50 µl of the OSC suspension no less than 30 min before the addition of oocytes to allow for culture equilibration (Fig. 1A). OSCs were co-cultured directly in suspension culture surrounding the denuded oocytes in the microdroplet under oil. IVM culture proceeded for 24 to 28 h, after which oocytes were removed from culture, imaged, and preserved for molecular analysis.

**Table 1:** Cell culture media conditions

Component	OSC-IVM	Media-IVM
IVM medium (Medicult, Cooper Surgical)	** + **	** + **
10 mg/ml HSA (Life Global, GHSA-125)	** + **	** + **
75 mIU/ml of recombinant FSH (Millipore, F4021)	** + **	** + **
100 mIU/ml of recombinant hCG (Sigma, CG10)	** + **	** + **
500 ng/ml androstenedione (Sigma, A-075 l)	** + **	** + **
1.0 µg/ml doxycycline (StemCell Tech., 100–1047)	** + **	** + **
100,000 OSCs per 100 µl droplet	** + **	**-**

In vitro maturation, *IVM*; human serum albumin, *HSA*; follicle stimulating hormone, *FSH*; human chorionic gonadotropin, *hCG*; ovarian support cells, *OSCs*; international units, *IU*

### In vitro maturation

All donated immature oocytes were maintained in preincubation LAG Medium (Medicult, Cooper Surgical) at 37 °C for 2–3 h after cumulus removal prior to introduction to in vitro maturation conditions for 24 to 28 h (either Media-IVM or OSC-IVM). Commercially available media for in vitro maturation were used as the base media for our studies (Medicult, Cooper Surgical). Although these media were designed for in vitro maturation of cumulus-enclosed oocytes, there are no commercially available media expressly designed for use with denuded oocytes following a retrieval employing an LH surge-mimicking trigger. Therefore, we have used media that we believe are the best available option for examining in vitro maturation of denuded immature oocytes.

### Experiment design

The purpose of this comparison was to determine whether the OSCs were the active ingredient or driver of oocyte maturation in the IVM co-culture system, defined as being able to positively influence nuclear maturation rate and oocyte transcriptomic profile. For this purpose, media in both experimental and control conditions were prepared by following the manufacturer’s recommendations and further supplemented with androstenedione and doxycycline (both necessary for activation of OSCs) in order to compare maturation outcomes with or without OSCs in the same medium formulation (see Table 1).

### Oocyte culture condition description

Donated oocytes were retrieved from 47 patients and pooled into 29 independent cultures, totaling 141 fresh oocytes, with 82 oocytes utilized in OSC-IVM (experimental) and 59 oocytes utilized in Media-IVM (control). Due to low and highly variable numbers of available immature oocytes from discarded oocyte donation, immature oocytes from each donor pool were distributed equitably between the two conditions when possible or into one condition at a time, with no more than 10 oocytes per culture. Specifically, immature oocytes (GV and MI) were cultured together and distributed as equally and randomly as possible between the two conditions. Immature oocytes were subjected to in vitro maturation at 37 °C for a total of 24–28 h in a tri-gas incubator with CO_2_ adjusted so that the pH of the bicarbonate-buffered medium was 7.2–7.3 and with the O_2_ level was maintained at 5%.

### Assessment of oocyte in vitro maturation

At the end of the in vitro culture, oocytes were harvested from culture dishes and mechanically washed of any residual OSCs. Oocytes were then individually assessed for maturation state according to the following criteria:

- GV—presence of a germinal vesicle, typically containing a single nucleolus within the oocyte.
- MI—absence of a germinal vesicle within the oocyte and absence of a polar body in the perivitelline space between the oocyte and the zona pellucida.
- MII—absence of a germinal vesicle within the oocyte and presence of a polar body in the perivitelline space between the oocyte and the zona pellucida.

Following assessment of in vitro maturation and morphology scoring, oocytes were individually imaged using digital photomicrography and, if required, examined by fluorescent imaging for the second meiotic metaphase spindle. No oocytes from this study were utilized or banked for fertilization, transfer, implantation, or reproductive purposes.

### Oocyte morphology scoring

Oocytes harvested post-IVM were individually imaged using digital photomicrography on the ECHO Revolve inverted fluorescence microscope using phase contrast imaging. The images were later scored according to the Total Oocyte Score (TOS) grading system [32]. A single trained embryologist was blinded, and oocytes were given a score of − 1, 0, 1 for each of the following criteria: morphology, cytoplasmic granularity, perivitelline space (PVS), zona pellucida (ZP) size, polar body (PB) size, and oocyte diameter. ZP and oocyte diameter were measured using ECHO Revolve Microscope software and the image analysis software FIJI (2.9.0/1.53t). The sum of all categories was taken to give each oocyte a total quality score, ranging from − 6 to + 6, with higher scores indicating better morphological quality. TOS grading was also performed on the cohort of IVF-MII oocytes as a control reference population.

### Examination of the second meiotic metaphase spindle and its position relative to the polar body

Previously vitrified denuded immature oocytes were thawed and equitably distributed across OSC-IVM and Media-IVM conditions before being cultured for 28 h. Additional donated previously vitrified MII oocytes were collected and stained to visualize the microtubules of the meiotic spindle apparatus by fluorescent microscopy as an IVF control (IVF-MII) (Fig. 3). MII oocytes were incubated in 2 µM of an alpha-tubulin dye (Abberior Live AF610) for 1 h in the presence of 10 µM verapamil (Abberior Live AF610). Spindle position was then visualized using fluorescent microscopy (ECHO Revolve microscope, TxRED filter block EX:560/40 EM:630/75 DM:585). The angle of the first polar body and spindle apparatus in the IVM oocytes was determined (with the vertex at the center of the oocyte) using FIJI software (Supplementary Fig. 2) [33]. This measurement was also made on the cohort of IVF-MII oocytes as a control reference population.

### Cryopreservation of oocytes and OSCs for subsequent molecular analyses

Following the completion of morphological examination, cells were individually placed in 0.2 ml tubes containing 5 µl Dulbecco’s Phosphate Buffered Saline (DPBS), frozen in liquid nitrogen, and were stored at − 80 °C until subsequent molecular analysis.

For experiments investigating changes in OSC gene expression, prior to cryopreservation, OSCs were cultured for 24 h in the presence or absence of denuded mouse immature oocytes.

### Single oocyte and OSC transcriptomics library preparation and RNA sequencing

Libraries for RNA sequencing were generated using the NEBNext Single Cell/Low Input RNA Library Prep Kit for Illumina (NEB #E6420) in conjunction with NEBNext Multiplex Oligos for Illumina (96 Unique Dual Index Primer Pairs) (NEB #E6440S), according to the manufacturer’s instructions. Briefly, cells or single oocytes frozen in 5 µl DPBS and stored at − 80 °C were thawed and lysed in lysis buffer, then RNA was processed for reverse transcriptase and template switching. cDNA was PCR amplified with 12–18 cycles, then size purified with KAPA Pure Beads (Roche). cDNA input was normalized across samples. Following fragmentation and end prep, NEBNext Unique Dual Index Primer Pair adapters were ligated, and samples were enriched using 8 cycles of PCR. Libraries were cleaned up with KAPA Pure Beads, quantified using Quant-iT PicoGreen dsDNA Assay Kit (Invitrogen), and then an equal amount of cDNA was pooled from each oocyte library. The pool was subjected to a final KAPA Pure bead size selection if required and quantified using Qubit dsDNA HS kit (Invitrogen). After verification of library size distribution (~ 325 bp peak) using Bioanalyzer HS DNA Kit (Agilent), the library pool was subjected to RNA sequencing analysis using the MiSeq Micro V2 (2 × 150 bp) or MiSeq V2 (2 × 150 bp) kit on an Illumina MiSeq according to the manufacturer’s instructions.

### Oocyte and OSC transcriptomics data analysis

Illumina sequencing files (bcl files) were converted into fastq read files using Illumina bcl2fastq (v2.20) software deployed through BaseSpace using standard parameters for low input RNA-seq of individual oocytes or OSC samples. Low input RNA-seq data gene transcript counts were aligned to *Homo sapiens* GRCH38 (v2.7.4a) genome using STAR (v2.7.10a) to generate gene count files and annotated using ENSEMBL [34]. Gene counts were combined into sample gene matrix files (h5). Computational analysis was performed using data structures and methods from the Scanpy (v1.9.1) package as a basis [35]. Gene transcript counts were normalized to 10,000 per sample and log (ln) plus 1 transformed. Principal component analysis was performed using Scanpy package methods focusing on 30 PCA components. Integration and project (batch) correction were performed using comBat [36]. Projection into two dimensions was performed using the Uniform Manifold Approximation and Projection (UMAP) method [37].

To define the expected transcriptomic profile for normal MII oocytes, we used the donated cohort of in vivo matured IVF-MII samples (*n* = 34) as a reference point. The IVF-MII Signature Score was calculated by applying the “rank genes function” in scanpy to generate the top 50 genes in this group. The GV fail-to-mature Signature Score was defined by calculating the differentially expressed genes between the IVF-MII oocytes and the GV oocytes from both the OSC-IVM and Media-IVM conditions. The top 50 differentially expressed genes in GVs were collected using both the Wilcoxon ranked sum test and the cosine similarity-based marker gene identification (COSG) method [38]. No other MI or MII oocyte sets were used as reference points, as these marker genes were developed to ensure minimal bias for other MII transcriptomic profiling. Cells were scored for similarity to each marker gene set using Scanpy gene marker scoring methods. To visualize our cells in signature marker space, we plotted the marker scores in two-dimensional space using the scatter plot function.

To explore the overall health and condition of the cells, we obtained gene signatures for several different pathways. To do so, we obtained gene lists for certain types of pathways from the Gene Set Enrichment Analysis (GSEA) database. Using these lists, we created signatures which we then plotted on a two-dimensional UMAP, as well as dotplots to show the expression across all conditions.

To compare the top genes among all the different conditions and maturation states, we used Scanpy to rank the genes among each group. For comparisons, we partitioned the data into three groups: OSC-IVM, Media-IVM, and IVF-MII. In each of the three groups, the top 50 genes were calculated and Venn diagrams were made to show which groups shared similar top genes. Functional enrichment analysis was performed using g:Profiler [39].

### Flow cytometry for OSC characterization

OSCs were incubated with a PE-conjugated mouse monoclonal antibody against CD82 (1:50 dilution; 342,104, BioLegend) in FACS wash (dPBS with 5% fetal bovine serum (FBS)). After incubation, cells were washed with FACS wash, stained with propidium iodide (1:20 dilution; P4864, Millipore Sigma) for live/dead cell staining, and subsequently analyzed using a CytoFlex Flow Cytometer. Unstained cells (negative controls) were used to determine the gating strategy.

### Data analysis and statistics

Oocyte maturation outcome data was analyzed using Python statistical packages pandas (1.5.0), scanpy (1.7.3), and statsmodels (0.13.2). Maturation percentages by donor group were analyzed using unpaired *t-*test as functions of the IVM environment as OSC-IVM or Media-IVM. *t*-test statistics were computed comparing OSC-IVM versus Media-IVM, then used to calculate *p*-values using Welch’s correction for unequal variance. One-way ANOVA was utilized for comparisons of more than two groups for spindle apparatus location analysis and oocyte gene expression score matrix, with follow-up multiple comparison testing. Bar graphs depict mean values for each population, and error bars represent the standard error of the mean (SEM). The number of independent oocytes for the experiment is indicated in the figure and Materials and Methods.

## Results

### hiPSC-derived OSCs effectively promote human oocyte maturation in co-culture with denuded oocytes

We have previously demonstrated that hiPSC-derived OSCs are predominantly composed of granulosa-like cells [30]. In response to FSH hormonal stimulation in vitro, the OSCs produce growth factors and steroids necessary for paracrine interaction with oocytes and cumulus cells [30]. Recently, we reported that hiPSC-derived OSCs are capable of significantly improving the rates of metaphase II (MII) and euploid blastocyst formation after abbreviated gonadotropin stimulation, demonstrating a novel approach to IVM with broad applicability to modern assisted reproductive technology (ART) practice [31]. In this study, we aimed to investigate whether the same hiPSC-derived OSCs could be used for rescuing immature denuded oocytes retrieved from standard COS cycles. For that, we leveraged the same protocol previously published to establish a co-culture system consisting of OSC cells with retrieved denuded immature oocytes and assessed maturation rates after 24–28 h (Fig. 1A, B) [31]**.**

Oocytes matured by OSC-IVM were compared to oocytes that spontaneously matured in the Media-IVM control, which contains the same culture medium and supplements, but no OSCs, and maturation rates were determined per oocyte culture group for each condition (Fig. 1C). Strikingly, we observed significant improvement in maturation outcome rates (~ 1.7X) for oocytes that underwent rescue IVM with OSCs (Fig. 1C). Specifically, the OSC-IVM group yielded a maturation rate of 62% ± 5.57% SEM versus 37% ± 8.96% SEM in the Media-IVM (Fig. 1C, p = 0.0138, unpaired *t-*test).

### hiPSC-derived OSCs express key hallmarks of human granulosa cells and display dynamic oocyte-tuned changes in gene expression

To further explore molecular mechanisms associated with this increase in maturation rates observed in the presence of OSCs, we performed bulk RNA-sequencing on OSCs prior to co-culture and after 24 h in media alone or in media containing oocytes (Supplementary Fig. 3A). As expected, OSCs cultured for 24 h in media alone are more closely related to OSCs processed prior to culture, suggesting that the support cells retain their transcriptomic signature after in vitro culture (Supplementary Fig. 3B). Interestingly, when OSCs are incubated with oocytes for the same period of time, we observe a shift in their hierarchical clustering, which becomes closer to primary cumulus cells. Functional profiling analysis of the genes upregulated in OSCs cultured in the presence of oocytes in comparison with OSCs cultured in media alone for 24 h is enriched for terms related to growth factors, such as “vascular endothelial growth factor receptor 2 binding,” “oxidoreduction-driven active transmembrane transporter activity,” “regulation of primary metabolic process,” “mitochondrial respirasome,” and “cytoplasm,” suggesting that OSCs are undergoing constant cellular remodeling when in the presence of oocytes (Supplementary Fig. 3C–D, Supplementary Table 1). Additionally, we assessed factors that are known to perform important roles in oocyte maturation and have been demonstrated to improve oocyte IVM (Supplementary Fig. 3B). As can be seen, OSCs upregulate expression of *CYP19A1*, a key enzyme in the steroidogenesis pathway (Supplementary Fig. 3B) [40–42]. Furthermore, our results show a high level of expression of *MDK*, a known growth factor that improves human oocyte IVM (Supplementary Fig. 3B) [43, 44]. We additionally find that expression of *IGF2*, *EGF*, and *IGFBP4* are high in both the 0 h and 24 h with oocyte cultures, suggesting oocyte co-culture maintains expression of these key growth factors known to promote oocyte IVM (Supplementary Fig. 3B) [4, 14, 45]. Conversely, we see high expression at 0 h and 24 h without oocytes of *EGFR* and *IGFBP2*, showing that oocyte co-culture downregulates these genes (Supplementary Fig. 3B) [46, 47]. We broadly find similarity in the expression of the genes in cumulus cells, suggesting a potential similar function of the OSCs and cumulus cells in the culture environment, but with some key differences in genes such as *MDK*, *EGF*, *and NR2F2*. This data suggests that paracrine factors secreted by oocytes may regulate OSC functionality during oocyte maturation and that the transcriptomic signature of OSCs can be also modulated to closely resemble primary cumulus cells.

### OSC-IVM leads to mature oocytes of comparable morphological features to oocytes matured in vivo and in vitro

**Fig. 2:**
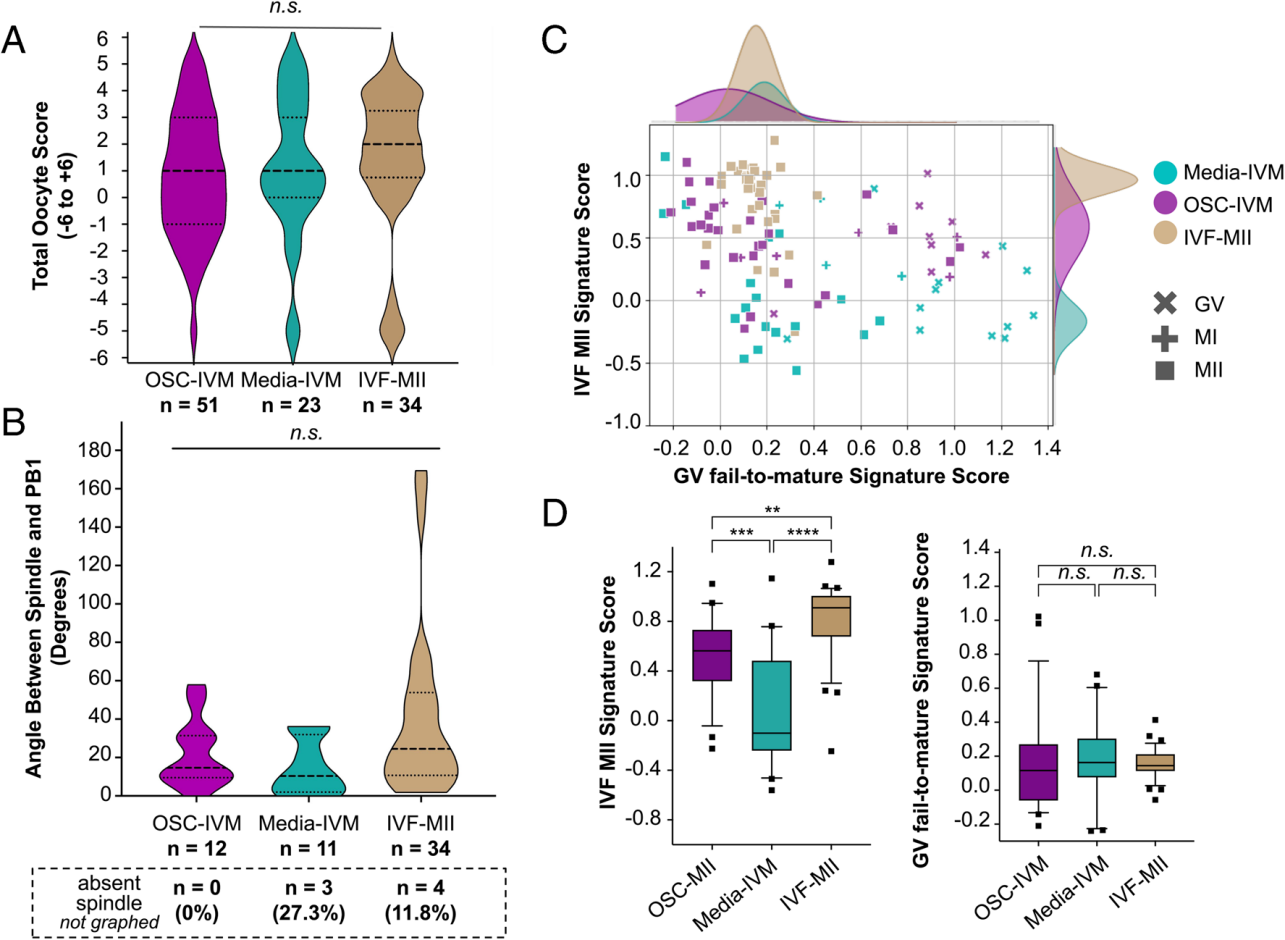
Transcriptomics analysis reveals that oocytes matured under the OSC-IVM condition are transcriptionally closer to IVF MII oocytes than those oocytes matured in Media-IVM, despite no changes in morphological features. **A** Total Oocyte Scores (TOS) generated from imaging analysis of MII oocytes after 24–28 h IVM experiments and IVF-MII oocytes. *n* indicates the number of individual MII oocytes analyzed. Median (dashed lines) and quartiles (dotted lines) are indicated. ANOVA indicated no significant (*p* = 0.5274) difference between the means of each condition. **B** Quantification of the angle between the first polar body (PB1) and spindle apparatus, derived from oocyte fluorescent imaging analysis (See Supplementary Fig. 2), of oocytes co-cultured with OSCs (OSC-IVM) or in media control (Media-IVM), and IVF-MII oocytes. *n* indicates the number of individual oocytes analyzed from each condition. Number and percentage (%) of MII oocytes with no spindle assembly observed are also indicated below the axis labels in the dashed box. Median (dashed line) and quartiles (dotted line) are indicated. ANOVA statistical analysis found no significant difference (ns, *p* = 0.1155) between the means of each condition. **C** Scatterplot projections of oocyte transcriptomes generated from the GV fail-to-mature Signature Score (X axis) and IVF MII Signature Score (Y axis). Symbols are color-coded based on the experimental condition (OSC-IVM, Media-IVM, IVF-MII), and symbol shapes represent oocyte maturation stages (GV, MI, and MII). Each symbol represents one oocyte. Histograms on top depict distribution of MII oocytes across the GV fail-to-mature Signature Score axis. Histograms on the right depict distribution of MII oocytes across the IVF MII Signature Score axis. Histograms are color-coded based on the experimental condition. *n* = 114 oocytes. **D** BoxPlot of distribution of MII oocytes across IVF MII Signature Score (left) and GV fail-to-mature Signature Score (right). ANOVA statistical analysis found a significant difference between the OSC-IVM-MII oocytes and Media-IVM MII oocytes (**p = 0.0003) and between the IVF-MII oocytes and Media-IVM oocytes (****p < 0.0001), as well as between the OSC-IVM-MII oocytes and the IVF-MII oocytes (**p < 0.0026)

We then sought to compare quality features between oocytes matured in vitro with the support of OSCs (OSC-IVM) against oocytes that had spontaneously matured in vitro (Media-IVM) or matured in vivo upon conventional gonadotropin stimulation cycle (IVF-MII control). We initially investigated morphological properties using Total Oocyte Score (TOS) analysis of the oocytes matured in vitro and in vivo and found no significant difference between the two groups (Fig. 2A, p = 0.5274, ANOVA), suggesting that addition of the OSCs during in vitro maturation of denuded oocytes does not affect the morphological features of MIIs.

To further evaluate oocyte quality, we additionally assessed the second meiotic spindle assembly. More specifically, we studied both the presence of and the angle of the spindle relative to the first polar body (PB1), which has been implicated in previous studies as a key indicator of oocyte quality relevant to fertilization and developmental competence, with a smaller angle indicating improved quality [33, 48]. We sought to determine the relative position of the second meiotic spindle apparatus and PB1 in OSC-treated oocytes (OSC-IVM) in comparison to control MII oocytes matured either in vitro (Media-IVM) or in vivo (IVF-MII) (Fig. 2B, Supplementary Fig. 2). We found that the spindle angle was not significantly different between conditions (MII OSC-IVM: 22° ± 5.2 SEM; MII Media-IVM: 15° ± 5.7 SEM; IVF-MII: 41° ± 8.3 SEM;* p* = 0.1155; ANOVA), suggesting that in vitro maturation of denuded oocytes that underwent IVM with either OSCs or conventional IVM Media alone does not impair spindle position. Interestingly, the only condition in which we did not observe instances of spindle absence was MII oocytes derived from OSC-IVM, even though the sample size was limited (Fig. 2B). More studies are needed to validate the relevance of this observation, but it is likely to indicate the formation of high-quality oocytes. Altogether, these results indicate that MII oocytes matured in vitro hold equivalent spindle angle values to MII oocytes directly retrieved from IVF procedures, suggesting that IVM applied to rescue denuded immature oocytes is not detrimental to oocyte quality based on these morphological parameters.

### MII oocytes matured in the presence of OSCs share a higher transcriptomic similarity to mature oocytes retrieved from conventional stimulation, compared to oocytes matured in IVM media

To further compare the quality and maturation of oocytes derived from OSC-IVM relative to a cohort of IVF-MII control oocytes and the Media-IVM oocytes, we performed a single oocyte transcriptomic analysis. Transcriptomic analyses provide a global view of oocyte gene expression and steady state transcript abundance, offering a strong representation of their cellular state, function, and general attributes. We started by combining our transcriptomic datasets that included (1) denuded immature oocytes after 24–28 h in co-culture with OSCs (OSC-IVM); (2) denuded immature oocytes cultured in the in vitro maturation media control (Media-IVM); and (3) MII oocytes retrieved from conventional IVF cycles (IVF-MII) (Supplementary Fig. 4A). We generated UMAP plots depicting individual oocytes by condition (OSC-IVM, Media-IVM, and IVF-MII) and maturation outcome (GV, MI, MII) (Supplementary Fig. 4A–C) and confirmed expression of oocyte markers (*DDX4* and *GDF9*; Supplementary Fig. 4D) and absence of granulosa and cumulus cell markers (*HAS2* and *CYP11A1*; Supplementary Fig. 4D). From this analysis, we observed that maturation state was the main driver of oocyte separation in whole transcriptomic space, suggesting that transcriptional profiles are a good predictor of oocyte maturation state. MII oocytes project predominantly into the large cluster on the lower right of the UMAP plot (green dots, Supplementary Fig. 4C), while GV oocytes project predominantly into a smaller cluster on the upper left of the UMAP plot (red dots, Supplementary Fig. 4C). In this UMAP representation, MII oocytes retrieved from IVF (IVF-MII) show close grouping together with MII from both the OSC-IVM, as well as Media-IVM (Supplementary Fig. 4B–C). Similarly, GVs (red dots) from OSC-IVM and Media-IVM were closely located and distinct from the MII oocytes (green dots) (Supplementary Fig. 4B-C). In contrast, MI oocytes (orange dots) were scattered across both groups, a likely consequence of their intermediate maturation state between GVs and MIIs (Supplementary Fig. 4B–C).

To further stratify our IVM oocytes by their transcriptional profile, we generated reference transcriptomic signatures for oocyte maturation outcomes. We used MII oocytes retrieved from conventional ovarian stimulation IVF samples (IVF-MII) to create a gene score for *IVF MII maturation signature* (Fig. 2C, Supplementary Table 2). In parallel, we used the stalled GVs resultant from IVM conditions (OSC-IVM and Media-IVM) to generate a gene score for *GV fail-to-mature signature* (Fig. 2C, Supplementary Table 2). These two gene signatures were utilized to capture a relative positive control of maturation, namely an IVF-like successful maturation outcome, as well as a negative control of maturation, namely oocytes that arrest as GVs. We then generated scatterplot projections to assess the molecular profile of individual oocytes relative to the *IVF MII maturation gene signature* (y-axis), as well as the *GV fail-to-mature gene signature* (x-axis) (Fig. 2C, Supplementary Table 2). For visual clarity, we annotated individual oocytes by condition (OSC-IVM, Media-IVM, and IVF-MII) and maturation outcome (GV, MI, MII) and included histograms to highlight the distribution of MII oocytes across both signature scores axes (Fig. 2C, Supplementary Table 2). As expected, we observed that most of the oocytes morphologically classified as GVs (“x” symbol) clustered in the lower right side of the plot, holding a high score for *GV fail-to-mature signature* along with a low score for *IVF MII maturation signature*. In contrast, individual oocytes from the IVF-MII condition clustered together in the upper left side of the plot, with a high score for *IVF MII maturation signature* and a low score for *GV fail-to-mature signature* (Fig. 2C, D). We then compared the *IVF MII Maturation Signature Score* of MII oocytes from both IVM conditions, OSC-IVM (score mean = 0.50 ± 0.06) and Media-IVM (score mean = 0.07 ± 0.10), to the IVF-MII oocytes (score mean = 0.81 ± 0.05) (Fig. 2D). Despite observing a significant difference in the *IVF MII Maturation Signature Score* between IVF-MII oocytes and those from either OSC-IVM (*p* = 0.0026) and Media-IVM MII oocytes (*p* < 0.0001), it is noteworthy that OSC-IVM MII oocytes exhibited a closer resemblance to the IVF-MII oocytes counterpart, suggesting a stronger transcriptomic similarity between these two groups (Fig. 2C, D). No significant differences were identified among the groups across the *GV fail-to-mature Signature Score* (Fig. 2C, D). Therefore, while no morphological differences were observed between oocytes that underwent IVM using either OSCs or conventional IVM Media alone, as shown in TOS and second meiotic spindle assembly studies (Fig. 2A, B), RNA sequencing revealed that transcriptomic profile of oocytes from these two groups does not reflect morphological similarities observed among all oocytes matured (Fig. 2C, D). Altogether, our data shows that MII oocytes derived from OSC-IVM condition were transcriptionally more similar to those from the IVF-MII condition than the MII oocytes matured in the Media-IVM control.

### Mature oocytes subjected to OSC-IVM exhibit enrichment of genes associated with both oocyte maturation and embryo development

**Fig. 3:**
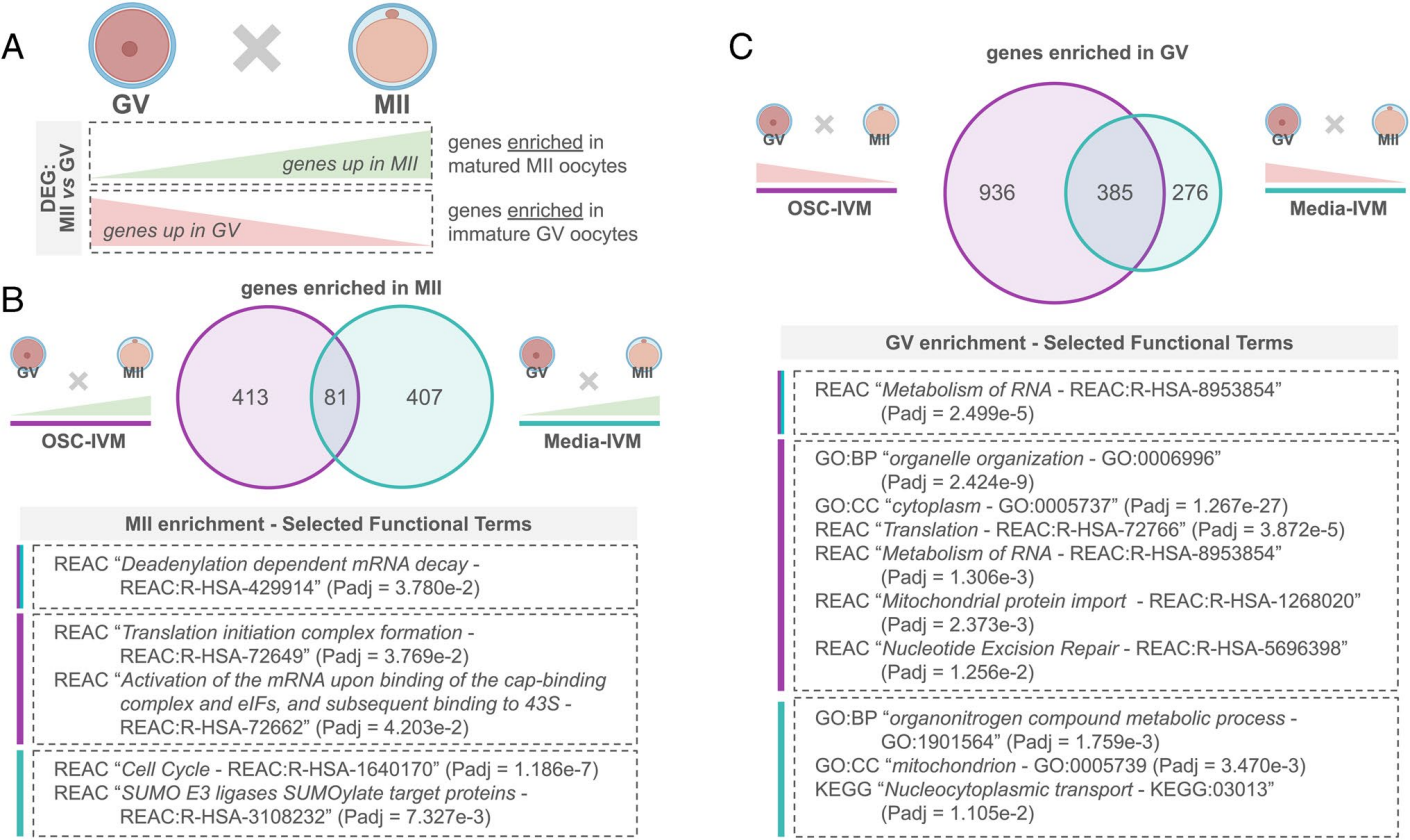
MII oocytes treated with OSC-IVM are enriched for genes related to oocyte maturation and embryo development. **A** Graphical illustration of the strategy to identify transcriptomic profile enrichment during the progression from the germinal vesicle (GV) to MII oocyte. Differentially expressed genes (DEG) were identified by comparing GVs to MII oocytes. **B** Venn diagram illustrating the gene enrichment in successfully matured MII versus fail-to-mature GVs treated with OSC-IVM versus Media-IVM oocytes. Numbers display the total number of genes identified. Selected functional enrichment analysis terms are shown for each subgroup. See Supplementary Fig. 5 for extended details. **C** Venn diagram illustrating the gene depletion in successfully matured MII versus fail-to-mature GVs treated with OSC-IVM versus Media-IVM oocytes. Numbers display the total number of genes identified. Selected functional enrichment analysis terms are shown for each subgroup. See Supplementary Fig. 5 for extended details

To better understand the transcriptomic differences among rescued in vitro matured MII oocytes subjected to either OSC-IVM culture or IVM media lacking OSCs (Media-IVM), we then analyzed the differentially expressed genes (DEGs) between successfully matured MII oocytes and fail-to-mature GV oocytes in both IVM conditions (Fig. 3A). From this analysis, we identified genes enriched or depleted in MII oocytes compared to fail-to-mature GVs for each condition. Comparison of MII-enriched genes from both conditions showed that OSC-IVM and Media-IVM MII oocytes share enrichment for genes involved in mRNA decay (Fig. 3B, C, Supplementary Table 3). Additionally, OSC-IVM MIIs displayed enrichment of genes involved in translation initiation and mRNA activation, implying that these MII oocytes may be acquiring the required proteins for the subsequent fertilization process. In contrast, Media-IVM MIIs displayed enrichment of genes involved in cell cycle, suggesting a potential alteration or stalling of the meiotic process of these mature oocytes (Fig. 3B, Supplementary Table 3). As anticipated, given that the oocyte genome is transcriptionally silent during maturation, there was a greater degree of transcript depletion rather than enrichment in MIIs compared to GVs (Fig. 3B, C, Supplementary Table 3). Furthermore, OSC-IVM MIIs displayed a higher number of significantly depleted transcripts compared to Media-IVM MIIs (1321 genes versus 661 genes), a further indication of a more in vivo-like maturation outcome as seen in other studies (Fig. 3C, Supplementary Table 4) [7, 49]. As expected, many of the MII-enriched genes in OSC-IVM encode for RNAs and proteins with relevant regulatory roles during oocyte maturation and embryonic development, such as *DAZL*, *TET2*, *WEE2*, *PRKAR2A*, *MSL3*, *PI4K2B*, *MBTD1*, and *CCP110* genes (Supplementary Fig. 5, Supplementary Table 4) [50–58]. We further mapped the top 50 marker genes expressed in MII oocytes matured in both OSC-IVM and Media-IVM and compared them to the in vivo control MII oocytes (IVF-MII) and find overall there is a high level of conservation among the top expressed transcripts (Supplementary Fig. 6, Supplementary Table 5). Altogether, these results indicate that interaction with OSCs might be modulating transcriptomic signatures of oocytes towards a profile more similar to conventional in vivo-like oocyte maturation. Therefore, these results support that oocytes matured in the presence of OSCs have a higher degree of molecular similarity to IVF-MII oocytes compared to their counterparts in the Media-IVM condition.

**Fig. 4:**
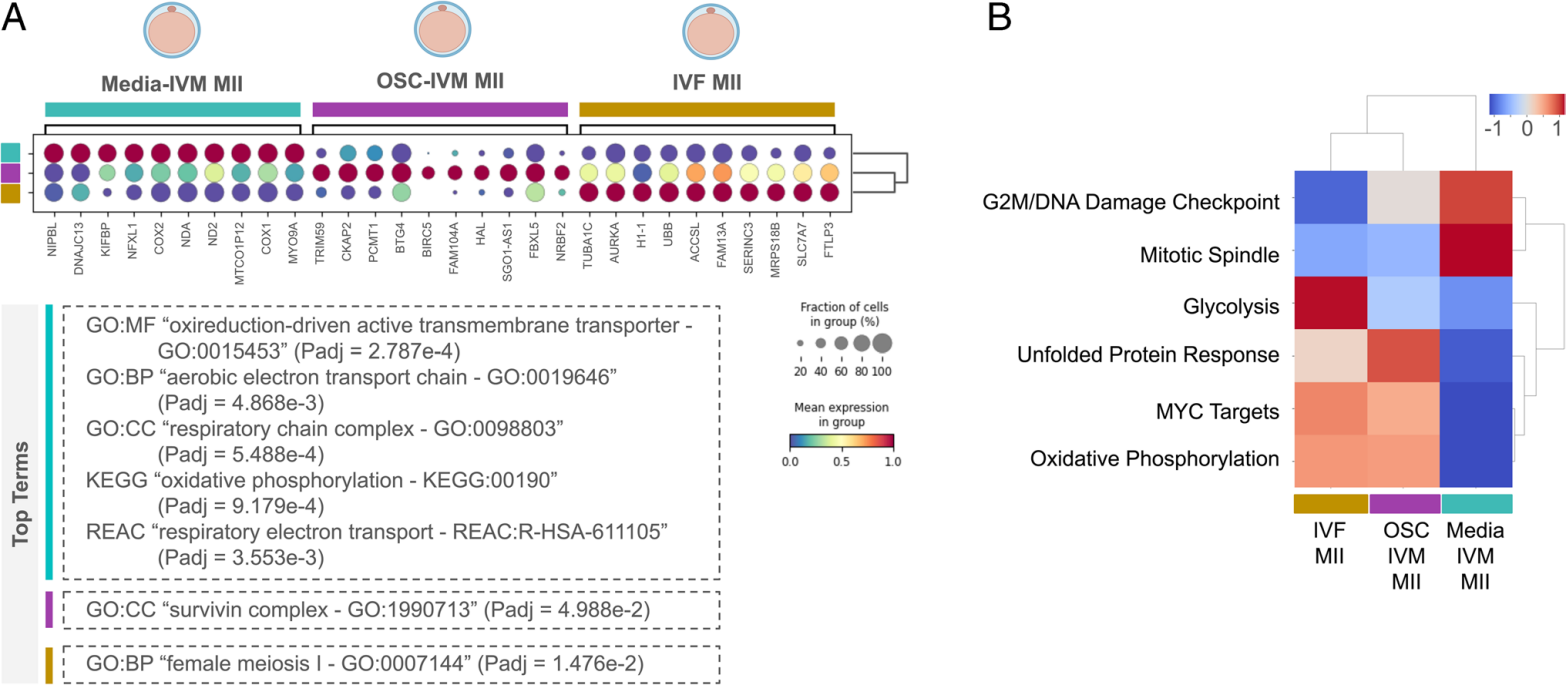
Pathway enrichment analysis reveals similarities between MII oocytes rescued from OSC-IVM and IVF-MII oocytes. **A** Dotplot displaying gene enrichment among Media-IVM MII oocytes, OSC-IVM MII oocytes, and IVF-MII oocytes. The bottom panel shows enrichment of Gene Ontology (GO) terms and KEGG and REACTOME pathways (see an extended version in Supplementary Fig. 7). GO, gene ontology; MF, molecular function; BP, biological process; CC, cellular component; KEGG, KEGG pathway; REAC, Reactome pathway. **B** Heatmap of Gene Set Enrichment Analysis (GSEA) hallmarks among Media-IVM MII oocytes, OSC-IVM MII oocytes, and IVF-MII oocytes. Heatmap represents row-normalized gene expression using a color gradients scale ranging from higher (red) to lower (blue) relative levels

After investigating differences between successfully matured MIIs and fail-to-mature GVs, we then compared transcriptional differences specifically in MII oocytes from the three groups (Fig. 4). We started by calculating DEGs in MII oocytes among all groups (Fig. 4A), as well as in between each of the two groups (Supplementary Fig. 7). Functional enrichment analysis further elucidated that genes overexpressed in oocytes subjected to OSC-IVM and IVF-MII oocytes were enriched for terms such as “survivin complex” and “female meiosis I,” respectively (Fig. 4A, Supplementary Fig. 8), being both of these processes critical for chromosome segregation during oocyte meiosis [59]. Conversely, oocytes subjected to the conventional media IVM condition overexpressed genes enriched for terms associated with the electron transport chain (ETC) and oxidative phosphorylation (OXPHOS) (Fig. 4A, Supplementary Fig. 8). While enhancing ETC and OXPHOS pathways could be beneficial for increasing cellular energy production, it could also elevate the generation of reactive oxygen species (ROS) and be a signal of cellular stress [60]. Taking a complementary approach, we leveraged Gene Set Enrichment Analysis (GSEA) and analyzed how each group of Hallmark genes was represented in each tested condition (Fig. 4B, Supplementary Fig. 9–12). Interestingly, key pathways involved in embryogenesis (Fig. 4B) were overrepresented in both IVF-MII as well as OSC-IVM groups, while Media-IVM MII oocytes displayed enrichment of DNA damage and spindle assembly markers [61, 62]. This indicates that the pathway enrichment profile in oocytes matured in the presence of OSCs is not only more similar to oocytes matured in vivo (IVF-MII), but also that the pathway enrichment profile shared by these two conditions may be associated with a higher developmental competence of these oocytes. Altogether, this data suggests that transcriptomic signature in oocytes is modulated by interaction with OSCs leading to the formation of MIIs sharing high transcriptomic similarity to oocytes matured in vivo (IVF-MII). These results highlight the potential of using this novel approach to rescue denuded immature oocytes from IVF procedures.

## Discussion

In this study, we demonstrate the use of hiPSC-derived OSCs for rescue in vitro maturation of human denuded oocytes (Fig. 1). These results demonstrate that OSC co-culture may be an effective platform for rescue maturation of immature denuded oocytes following conventional ovarian stimulation. Our work is the first of its kind to explore the potential of allogeneic stem cell-derived OSCs as a tool for rescue IVM of denuded oocytes in humans and shows the value of this and other cell engineering approaches for improving ART treatments and patient success outcomes.

It is generally a concern that IVM can result in oocytes of poor morphological quality and with gene expression profiles that are significantly different from conventional IVF oocytes [63]. Our measures of morphology indicate that there is no striking difference in the morphological quality between IVM conditions and IVF and no significant difference in the angle between the spindle and PB1 compared to IVF MIIs (Fig. 2A, B). However, it should be noted that the angle between the spindle and PB1 in the IVF group could be broadened because the already-present PB1 can be displaced during removal of the cumulus, whereas this does not occur when cumulus is removed from oocytes prior to culture for IVM and prior to PB1 extrusion.

Although we did not report any morphological differences among rescue IVM oocytes and IVF-MII control, our molecular analysis revealed that OSC-IVM MII oocytes had gene expression signatures that were more similar to IVF-MII controls than MII oocytes from Media-IVM. We show that a subset of oocytes that are morphologically MIIs can show an “under-matured” transcriptomic profile, particularly in the Media-IVM condition, where the vast majority of MII oocytes showed a low IVF signature score. This finding is notable with clinical implications, as it is established that rescue IVM oocytes have lower developmental competence than their in vivo matured sibling oocytes. Rescue-IVM inherently represents a non-ideal IVM context in which the oocytes often lack cumulus enclosures and have failed to mature after conventional stimulation, which may indicate a lack of developmental competence. Nonetheless, our data suggests the use of OSC-IVM can improve not only the nuclear maturation of these oocytes but also the cytoplasmic maturation, as measured by gene expression profile, yielding more IVF-like MII oocytes compared to more conventional rescue IVM approaches that rely on media alone.

Moreover, we demonstrated that genes differentially expressed in MII oocytes compared to fail-to-mature GVs in the OSC-IVM group share broad similarity to IVF-MII oocytes and show enrichment for terms and pathways associated with oocyte maturation and embryo development. The gene analysis tools applied in this study could not discern whether this gene enrichment observed in successfully matured MIIs in comparison with fail-to-mature GVs is due to targeted transcript degradation in GVs, minor gene upregulation, or more likely via post-transcriptional processing of transcripts rendering them more detectable in mature oocytes, or a combination of all three. It is well-described that oocytes undergo global transcriptomic silencing and selective degradation of maternal transcripts during the maturation process [6, 64]. A number of studies have likewise identified transcripts that are “exclusively” present in MII oocytes and not GV oocytes, despite the near complete transcriptional silencing that accompanies maturation [7, 8]. Our findings were largely in line with those studies, showing a greater number of depleted transcripts in the MII state compared to enriched transcripts, with OSC-IVM MIIs showing a greater overall depletion compared to Media-IVM. These data suggest that OSC-IVM rescued oocytes may have an improved degree of cytoplasmic maturation compared to oocytes matured in the Media-IVM condition, which is an important aspect for measuring the clinical utility of these rescued oocytes. However, despite the transcriptomic analysis indicating a potential enhancement in oocyte cytoplasmic maturation following OSC-IVM, further analyses assessing oocyte functional competence are required to corroborate this observation. Additionally, oocytes matured in co-culture with OSCs also showed minimal evidence of gene expression pathway signatures involved in DNA damage response, DNA repair, or oxidative stress, indicative of good health.

While elucidation of the mechanism of action of OSCs to improve oocyte rescue maturation rate and its influence on oocyte gene expression signatures is not within the scope of this study, the promising results warrant further mechanistic research. In our analysis, the OSCs show dynamic changes in gene expression overtime that are influenced by oocyte presence in the co-culture, hinting at active paracrine signaling in a non-static environment. It has been well documented that secretion of GDF9 and BMP15 by oocytes has the capability to reprogram surrounding granulosa cell environments in vitro, and our findings suggest that the OSCs are permissive to changing cell state in response to oocyte signaling [42, 65, 66]. We further note the dynamic and oocyte-tuned expression of key growth factors, enzymes, and receptors such as *EGF*, *IGF2*, *MDK*, *CYP19A1*, *IGFBP4*, *AMHR2*, and *FSHR* suggesting coordinated bidirectional cross talk between the oocytes and OSCs in the culture setting. Future studies focusing on causally linking secreted growth factors, steroids, and metabolites produced by the OSCs to changes in oocyte maturation may uncover the diverse and complex regulatory role of these cells on oocyte maturation.

The method of co-culture IVM described here integrates readily with existing procedures in ART laboratories, requiring no special equipment that is not commonly found in ART laboratories and minimal training of clinical embryology staff. Further, we demonstrate that the OSC-IVM platform can be applied to mature denuded oocytes from hCG-triggered cycles. Additionally, the primarily paracrine interaction between the oocytes and OSCs in this system, in which direct connections and gap junctions are not formed and soluble factors are exchanged, allows for a broad range of potential culture configurations. The applicability to denuded oocytes provides an expansion of functionality over other IVM systems that require that COCs be left either partially or entirely intact. This is particularly important as modern IVF practice more often adopts intracytoplasmic sperm injection (ICSI) and oocyte cryopreservation, which requires denudation of oocytes at retrieval.

IVM remains a clinically important tool for reducing hormonal burden, expanding ART access, and providing treatment to patients who cannot or do not wish to undergo conventional IVF due to medical contraindication. Further studies that build on our understanding of the requisite developmental niche of human oocytes in vitro and use those findings to improve culture conditions will continue to improve IVM efficacy and provide better treatment options for a diverse range of patient demographics, treatment styles, and oocyte dispositions.

## Limitations

While the number of subjects and oocytes utilized in this study was limited, our findings represent an important step in establishing stem cell-derived OSCs as a co-culture platform for rescue IVM and understanding how oocyte developmental quality is influenced by the OSC-IVM environment. An inherent limitation of rescue IVM studies is that the number of oocytes available to rescue from a donor is highly variable, making equal distribution challenging. A prospective study evaluating the maturation rates of rescue OSC-IVM and other rescue IVM approaches is warranted to further validate these findings. Furthermore, the limited sample size for spindle analysis and use of cryo-preserved (vitrified and slow-frozen) oocytes in that analysis and the live cell-based imaging method warrants a larger sample size and potentially a fixed cell, immunofluorescence imaging-based approach to validate findings and provide more granular analysis of spindle health such as shape and orientation as well as chromosomal segregation dynamics. An additional limitation of this study is the inclusion of differently treated oocytes (fresh, vitrified, slow-frozen, and snap-frozen), which may introduce heterogeneity in oocyte condition. To mitigate this variability, we conducted assays using either fresh oocytes (for maturation rate assessment) or cryopreserved oocytes (vitrified or slow-frozen for spindle analysis, and snap-frozen for RNA-seq), and we followed randomized sibling oocyte allocation. Furthermore, another factor contributing to a potential variability is the use of oocytes obtained from various fertility centers, following different ovarian stimulation regimens, alongside the inclusion of individuals from different ages. Although we recognize that oocyte characteristics might be influenced by these diverse treatment modalities, our RNAseq data and spindle assembly analysis did not reveal any significant association based on oocyte status or other factors such as stimulation regimen, clinical provider, or donor characteristics, which reinforces the robustness of our results. Additionally, an inherent limitation in utilizing RNA-sequencing is that the oocyte is destroyed in the analysis, making definitive links between developmental competence such as embryo formation and transcriptomic signature challenging. More research is needed to determine the embryo formation capacity of these rescue OSC-IVM oocytes, as well as the epigenetic health of embryos rescued via OSC-IVM. In addition, future studies will determine if rescue OSC-IVM embryos are capable of healthy implantation, development, and live birth compared to traditional rescue IVM and IVF controls. Regardless of the limitations, the use of OSCs derived from hiPSCs represents a novel approach for reproducible, highly standardized delivery of ovarian support cell co-culture in IVF and IVM settings, a key practical consideration for use in a clinical treatment setting. Additionally, the use of a single source of OSCs allows for consistent production that does not require per patient customization, avoiding the challenges of autologous and primary cell heterogeneity and ensuring this approach can be widely utilized in standard clinical practice.

### Supplementary Information

Below is the link to the electronic supplementary material.

Supplementary file1 (XLSX 908 KB)

Supplementary file2 (XLSX 8 KB)

Supplementary file3 (XLSX 2601 KB)

Supplementary file4 (XLSX 612 KB)

Supplementary file5 (XLSX 11 KB)

Supplementary file6 (JPG 2180 KB)

Supplementary file7 (JPG 1497 KB)

Supplementary file8 (JPG 3472 KB)

Supplementary file9 (JPG 3022 KB)

Supplementary file10 (JPG 4675 KB)

Supplementary file11 (JPG 2685 KB)

Supplementary file12 (JPG 2048 KB)

Supplementary file13 (JPG 2461 KB)

Supplementary file14 (JPG 1194 KB)

Supplementary file15 (JPG 3746 KB)

Supplementary file16 (JPG 2970 KB)

Supplementary file17 (JPG 1935 KB)

## Data Availability

All data needed to evaluate the conclusions in the paper are present in the paper and supplementary tables and figures. Raw and processed sequencing data will be deposited to GEO upon publication. Anonymized raw data for all findings in the paper will be provided upon request.
