# Supplementary figures and images for: Rescue in vitro maturation using ovarian support cells of human oocytes from conventional stimulation cycles yields oocytes with improved nuclear maturation and transcriptomic resemblance to in vivo matured oocytes
Source: J Assist Reprod Genet. 2024 May 30;41(8):2021–36. doi: 10.1007/s10815-024-03143-4 (PMC11339229; doi:10.1007/s10815-024-03143-4)

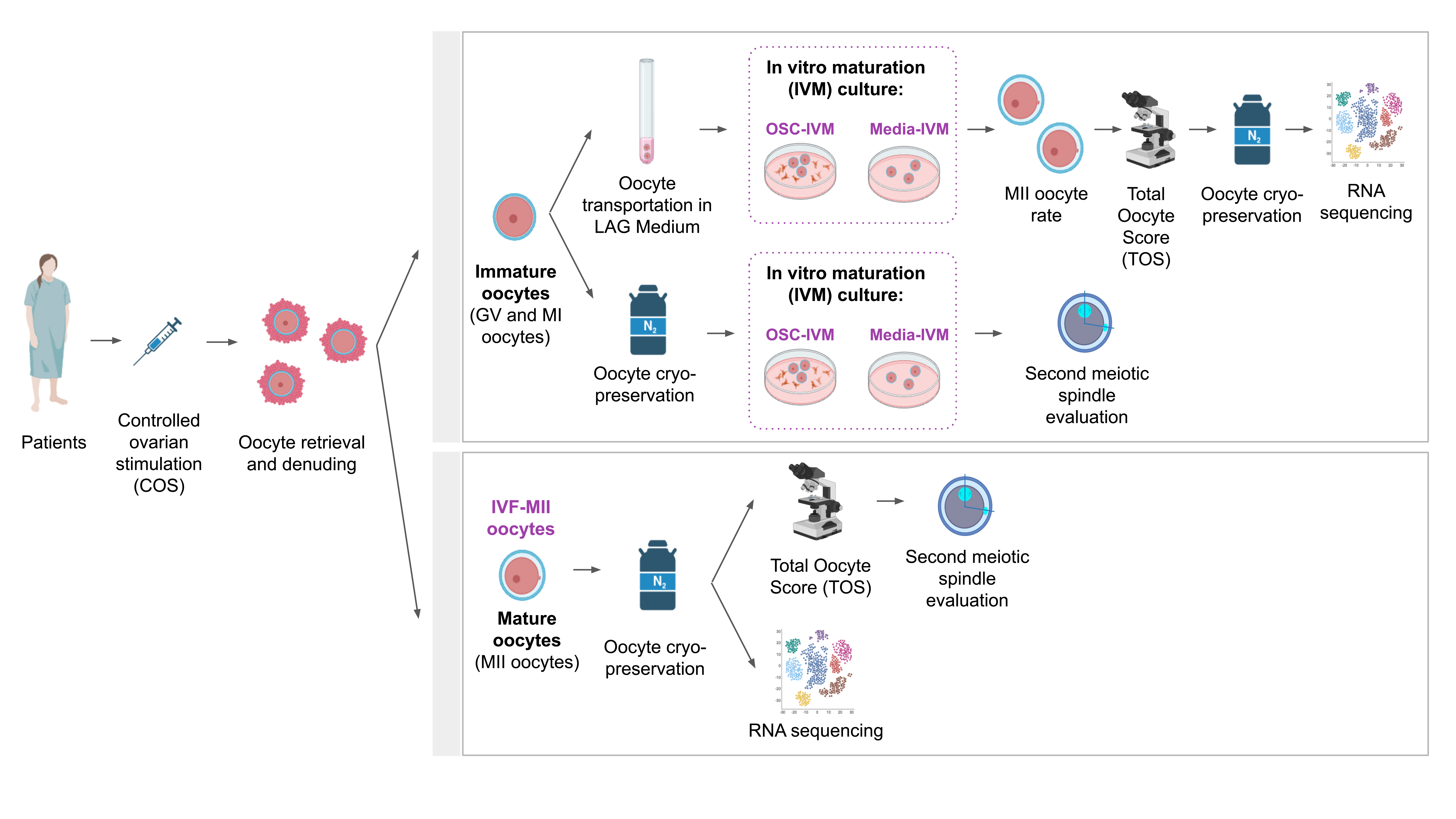

Supplement: Supplementary file 6 — Supplementary file6 (JPG 2180 KB) [file 10815_2024_3143_MOESM6_ESM.jpg]

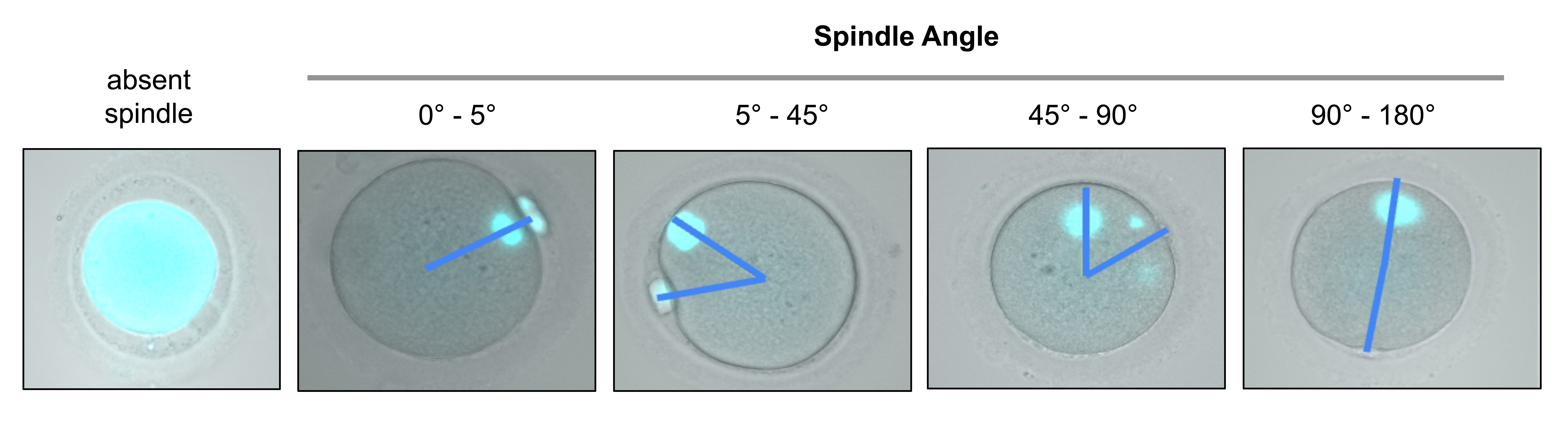

Supplement: Supplementary file 7 — Supplementary file7 (JPG 1497 KB) [file 10815_2024_3143_MOESM7_ESM.jpg]

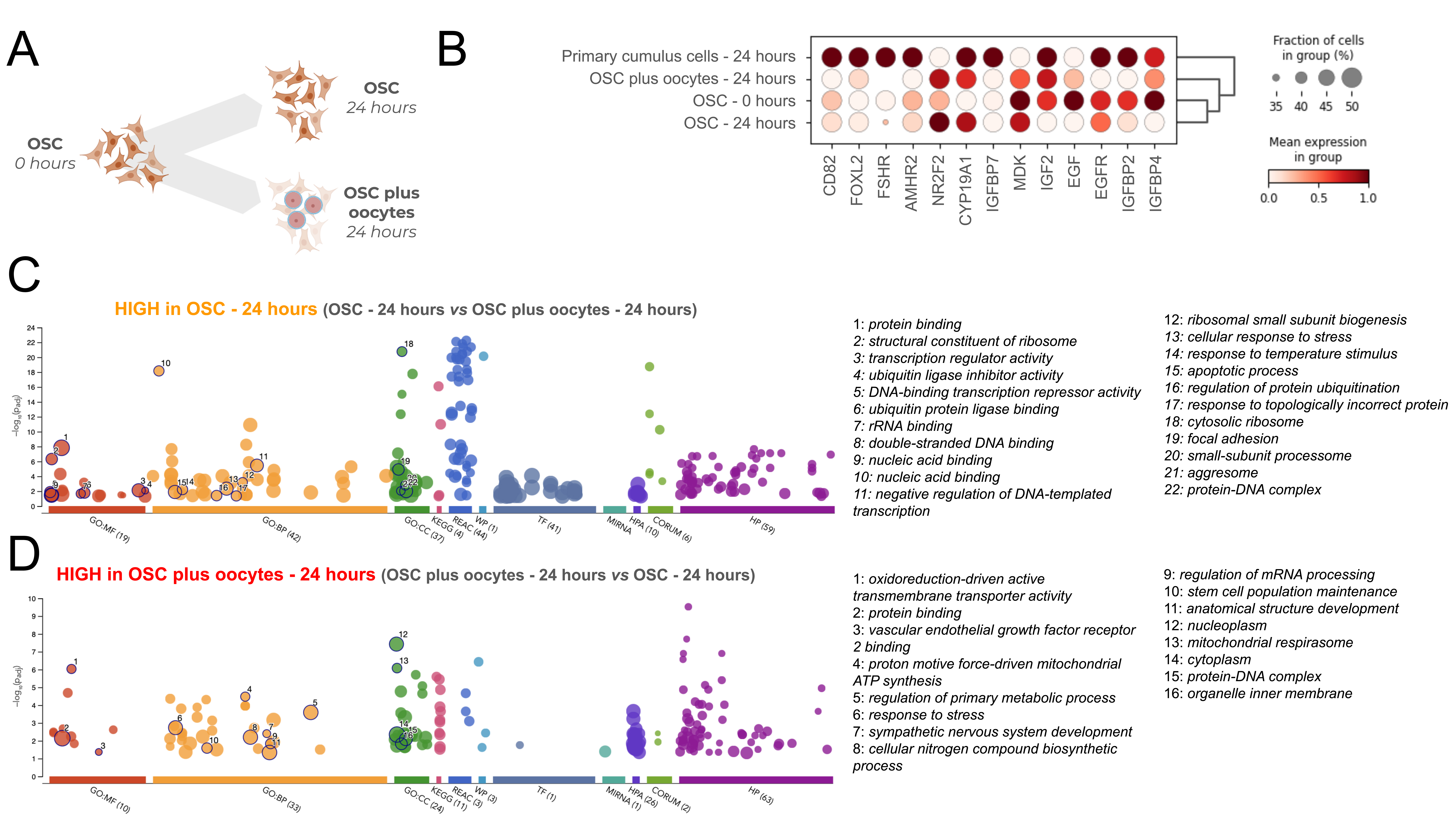

Supplement: Supplementary file 8 — Supplementary file8 (JPG 3472 KB) [file 10815_2024_3143_MOESM8_ESM.jpg]

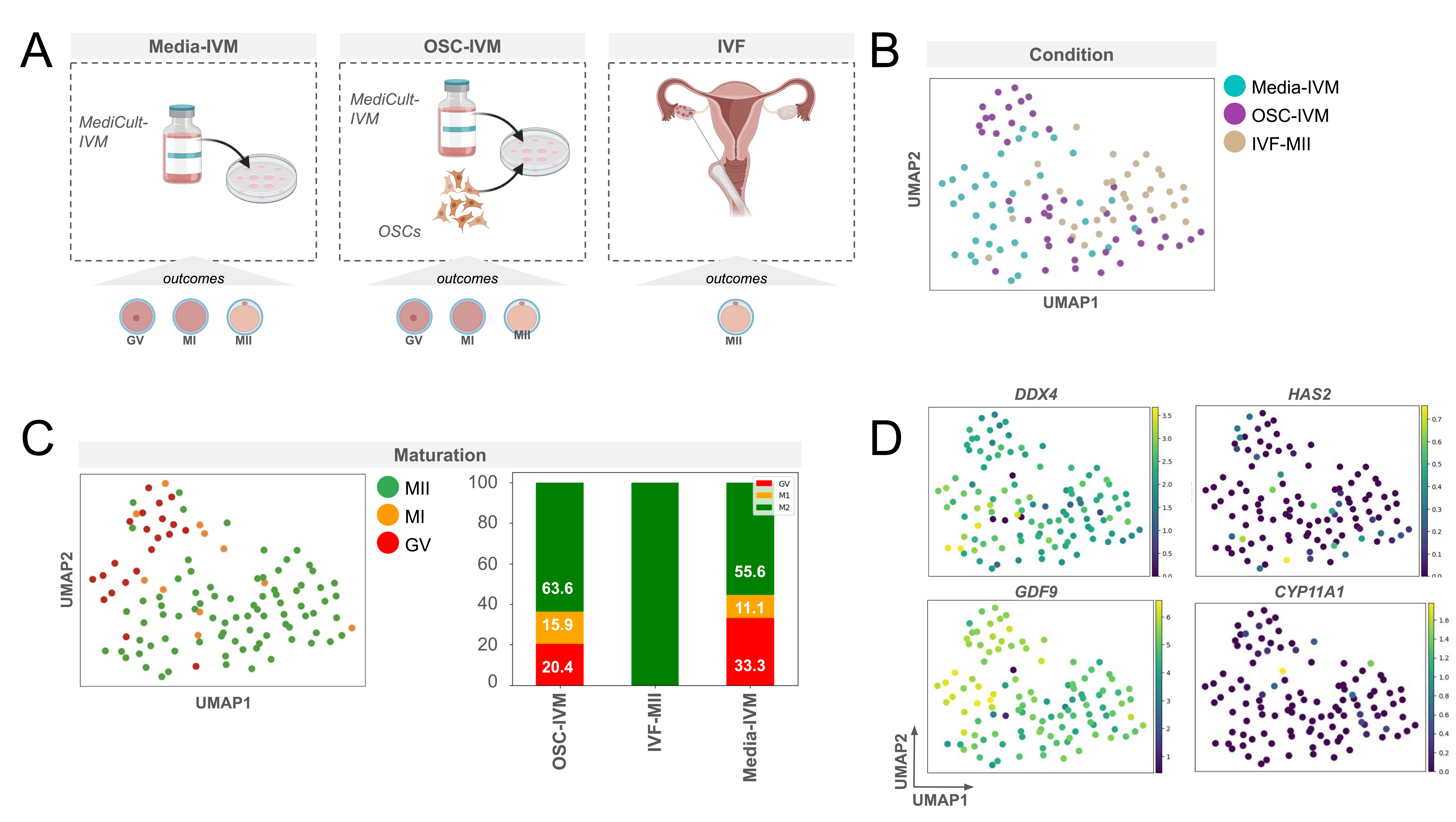

Supplement: Supplementary file 9 — Supplementary file9 (JPG 3022 KB) [file 10815_2024_3143_MOESM9_ESM.jpg]

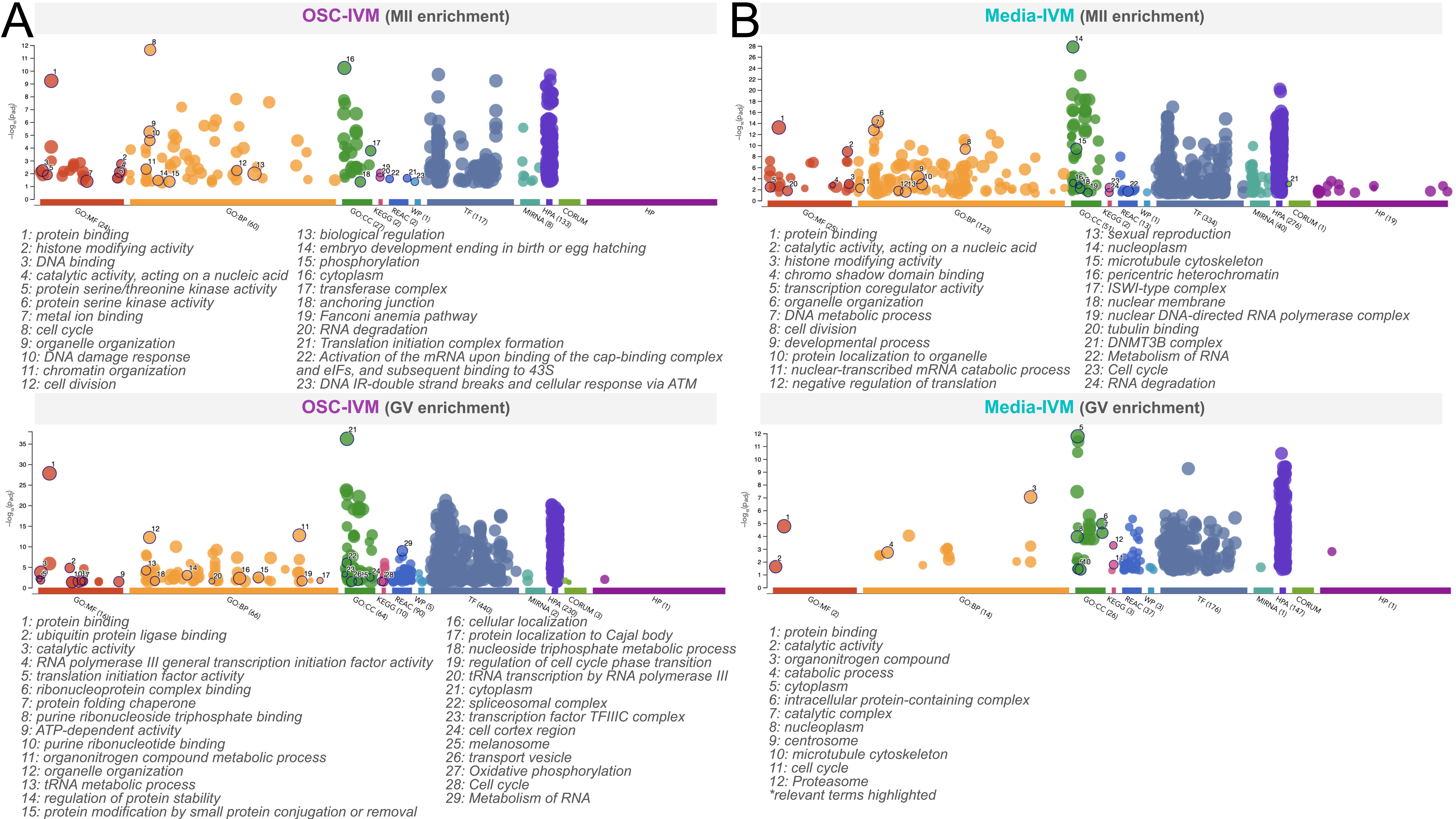

Supplement: Supplementary file 10 — Supplementary file10 (JPG 4675 KB) [file 10815_2024_3143_MOESM10_ESM.jpg]

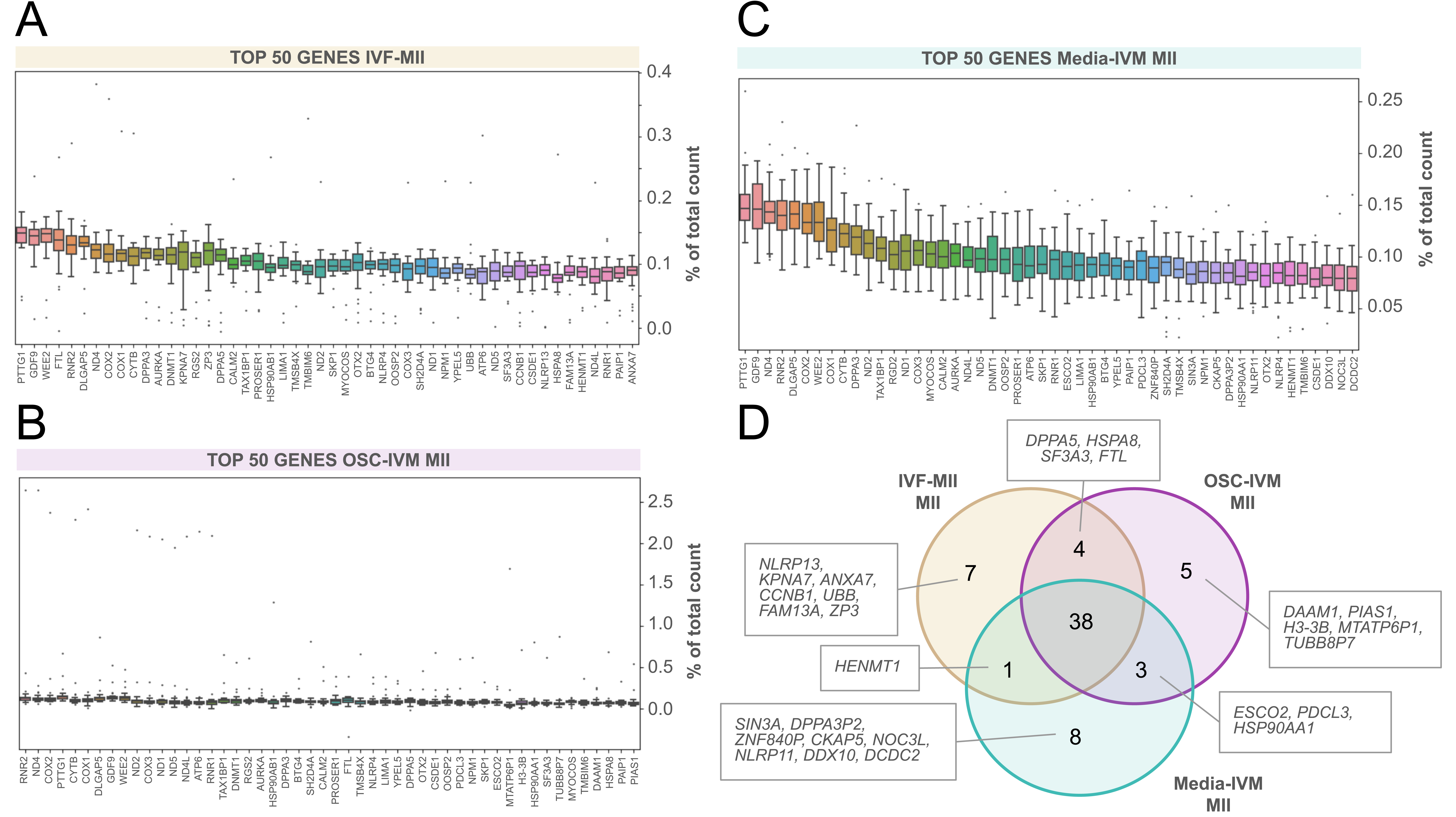

Supplement: Supplementary file 11 — Supplementary file11 (JPG 2685 KB) [file 10815_2024_3143_MOESM11_ESM.jpg]

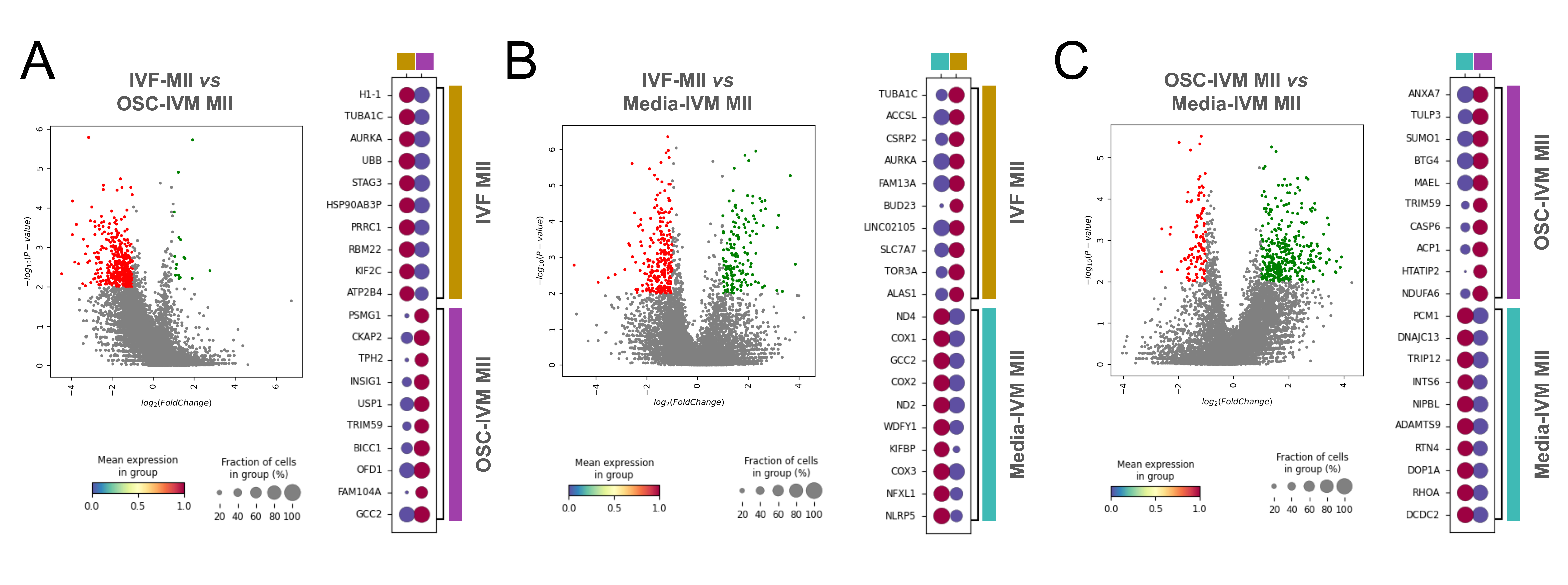

Supplement: Supplementary file 12 — Supplementary file12 (JPG 2048 KB) [file 10815_2024_3143_MOESM12_ESM.jpg]

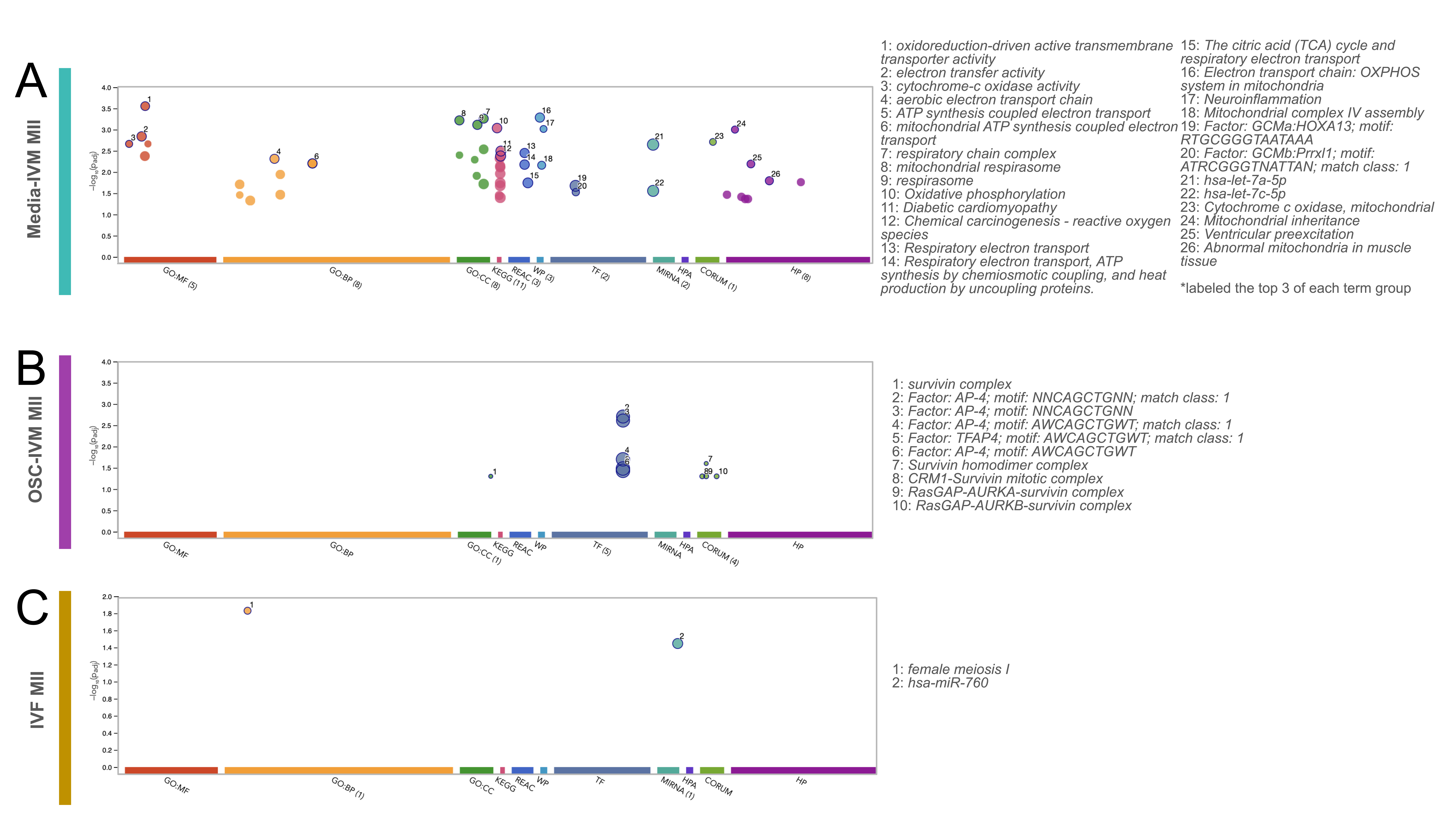

Supplement: Supplementary file 13 — Supplementary file13 (JPG 2461 KB) [file 10815_2024_3143_MOESM13_ESM.jpg]

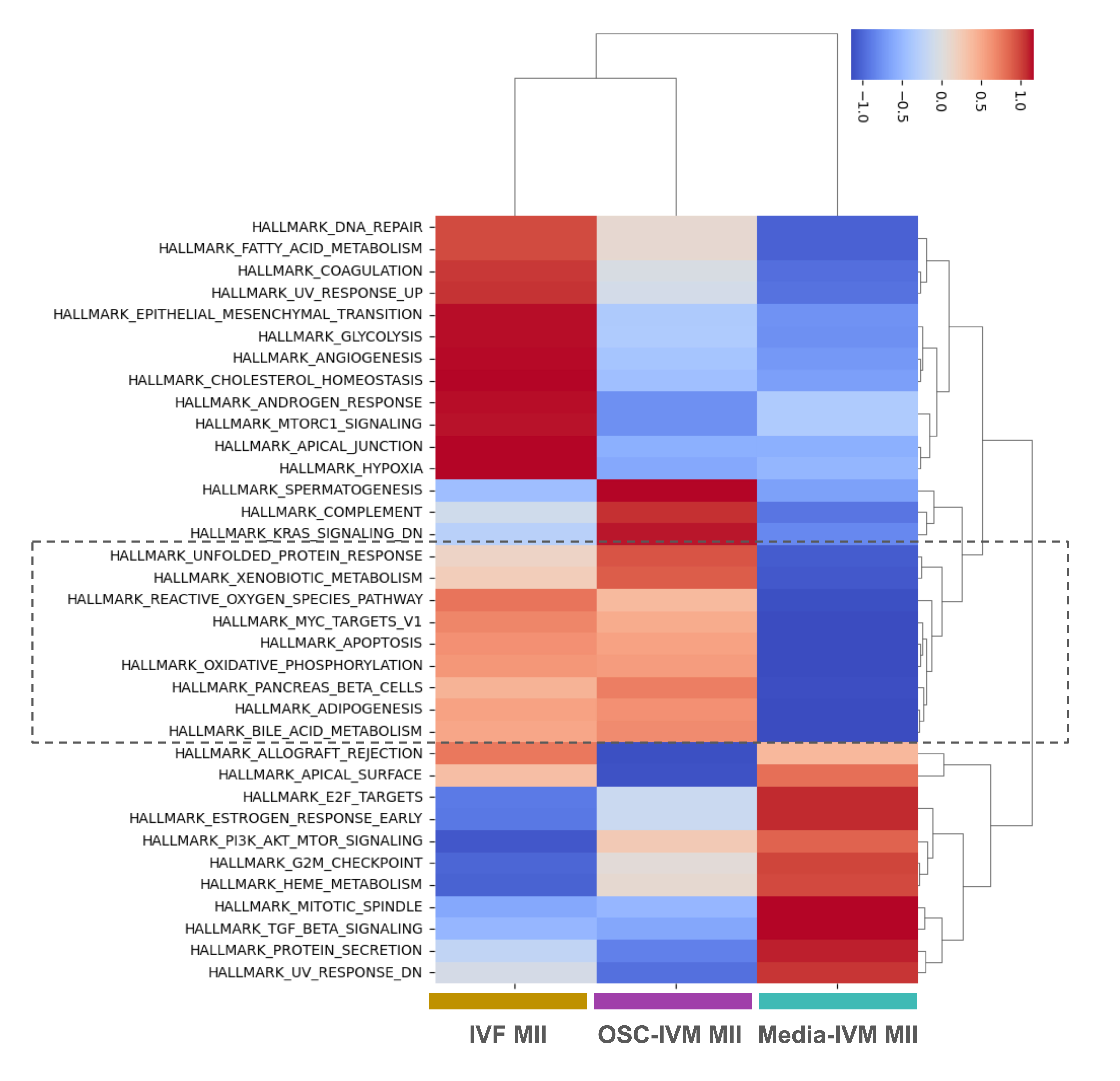

Supplement: Supplementary file 14 — Supplementary file14 (JPG 1194 KB) [file 10815_2024_3143_MOESM14_ESM.jpg]

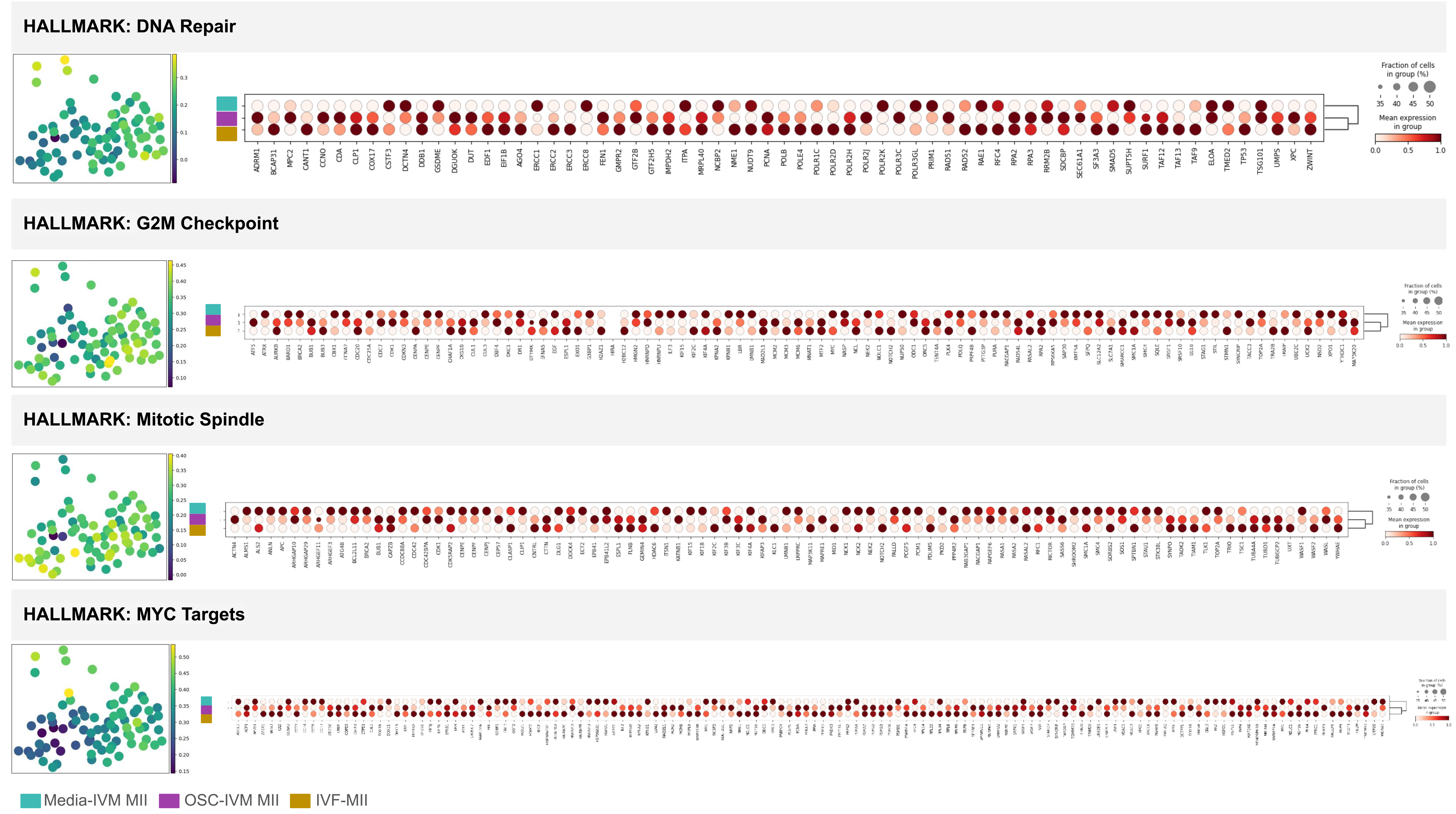

Supplement: Supplementary file 15 — Supplementary file15 (JPG 3746 KB) [file 10815_2024_3143_MOESM15_ESM.jpg]

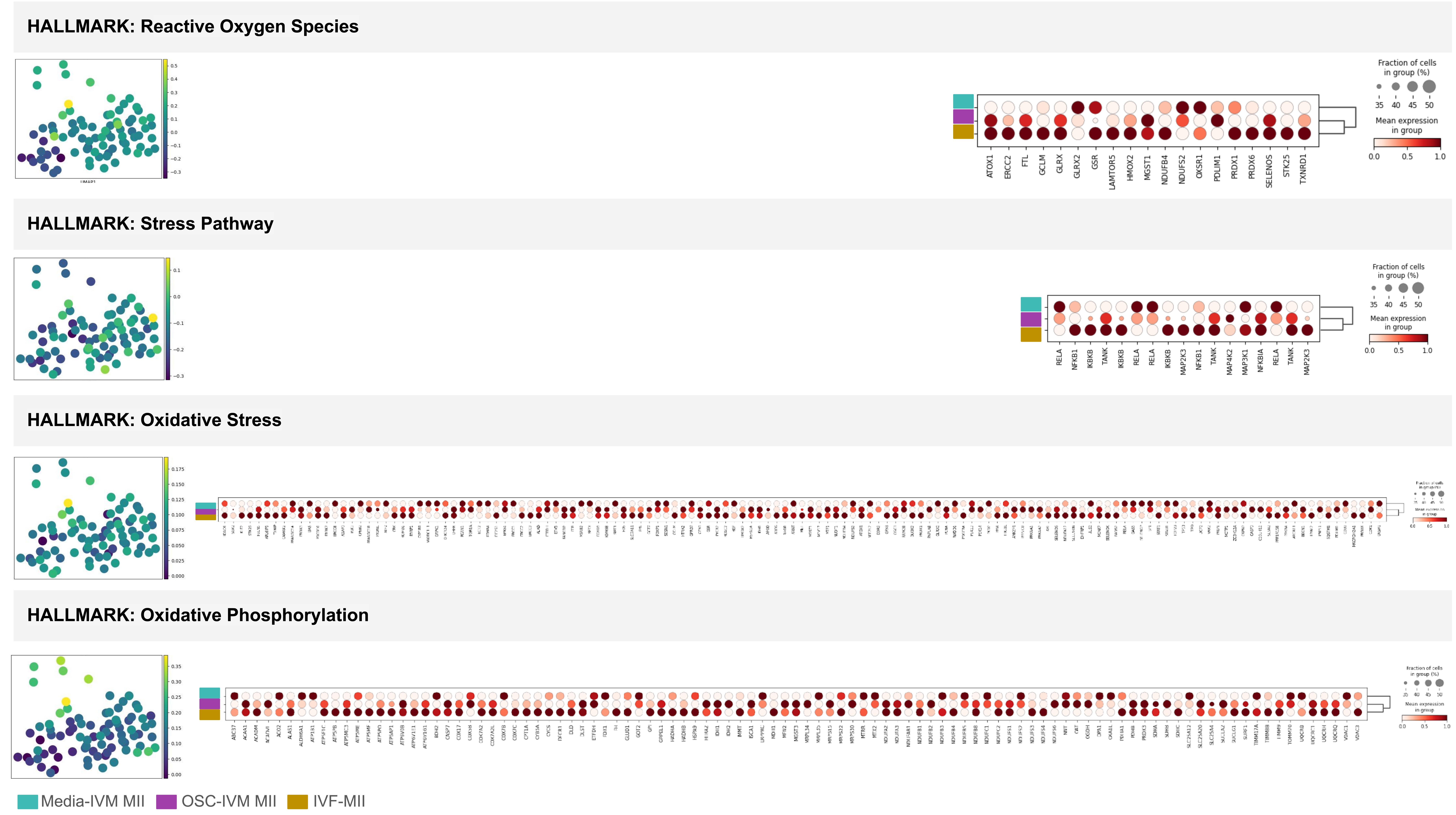

Supplement: Supplementary file 16 — Supplementary file16 (JPG 2970 KB) [file 10815_2024_3143_MOESM16_ESM.jpg]

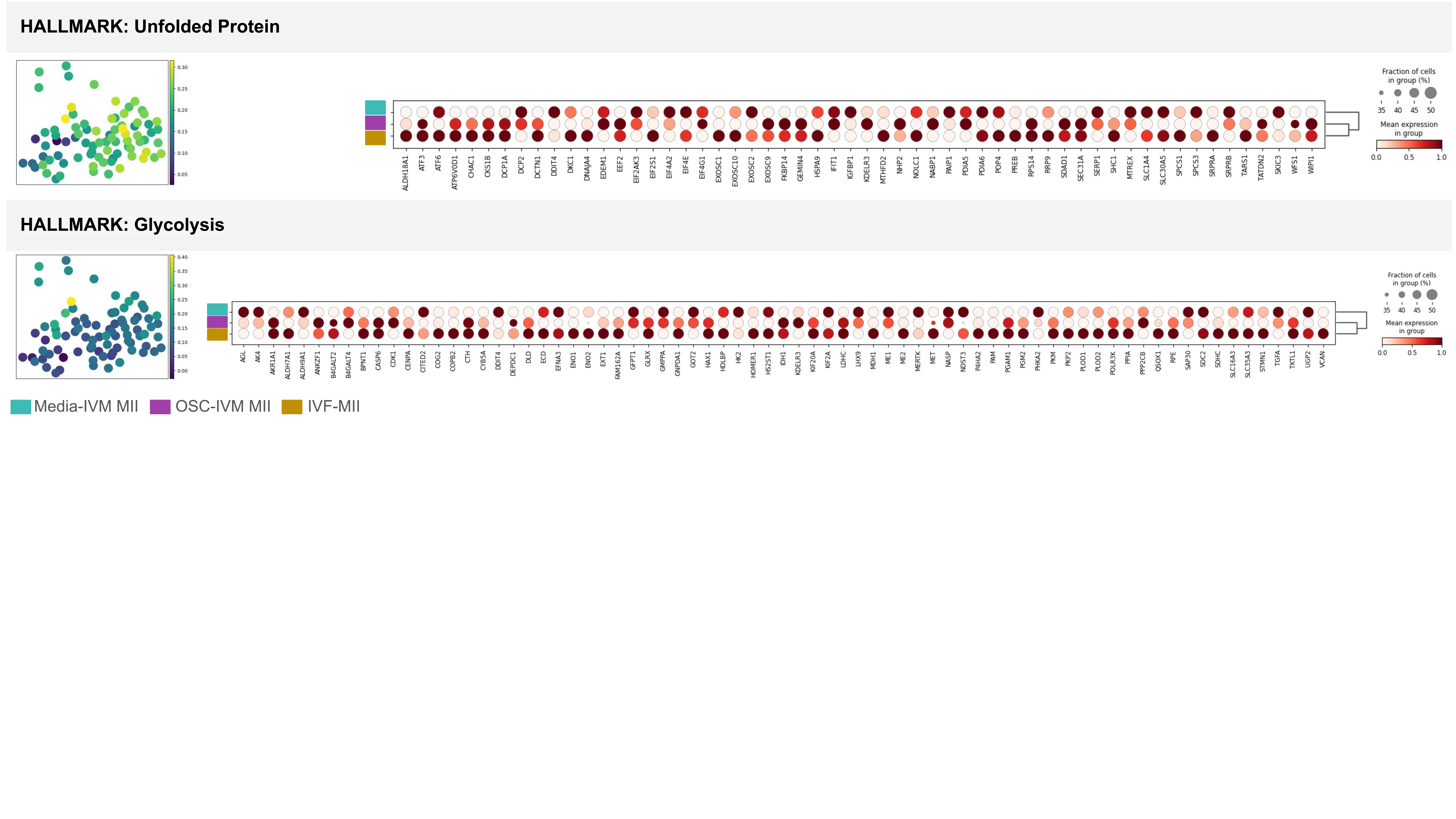

Supplement: Supplementary file 17 — Supplementary file17 (JPG 1935 KB) [file 10815_2024_3143_MOESM17_ESM.jpg]
